# Single-Component Color-Tunable Smart Organic Emitters with Simultaneous Multistage Stimuli-Responsiveness and Multimode Emissions

**DOI:** 10.34133/research.0241

**Published:** 2023-09-28

**Authors:** Yu Yan, Chengfang Liu, Jianzhong Fan, Yusheng Li, Huanling Liu, Qian Wang, Xiangchun Li, Junfeng Li, Wen-Yong Lai

**Affiliations:** ^1^ State Key Laboratory of Organic Electronics and Information Displays (SKLOEID), Institute of Advanced Materials (IAM), School of Chemistry and Life Sciences, Nanjing University of Posts & Telecommunications, 9 Wenyuan Road, Nanjing 210023, China.; ^2^ Shandong Province Key Laboratory of Medical Physics and Image Processing Technology, School of Physics and Electronics, Shandong Normal University, Jinan 250358, China.

## Abstract

Achieving color-tunable emission in single-component organic emitters with multistage stimuli-responsiveness is of vital significance for intelligent optoelectronic applications, but remains enormously challenging. Herein, we present an unprecedented example of a color-tunable single-component smart organic emitter (DDOP) that simultaneously exhibits multistage stimuli-responsiveness and multimode emissions. DDOP based on a highly twisted amide-bridged donor–acceptor–donor structure has been found to facilitate intersystem crossing, form multimode emissions, and generate multiple emissive species with multistage stimuli-responsiveness. DDOP pristine crystalline powders exhibit abnormal excitation-dependent emissions from a monomer-dominated blue emission centered at 470 nm to a dimer-dominated yellow emission centered at 550 nm through decreasing the ultraviolet (UV) excitation wavelengths, whereas DDOP single crystals show a wide emission band with a main emission peak at 585 nm when excited at different wavelengths. The emission behaviors of pristine crystalline powders and single crystals are different, demonstrating emission features that are closely related to the aggregation states. The work has developed color-tunable single-component organic emitters with simultaneous multistage stimuli-responsiveness and multimode emissions, which is vital for expanding intelligent optoelectronic applications, including multilevel information encryption, multicolor emissive patterns, and visual monitoring of UV wavelengths*.*

## Introduction

Organic emitters have been extensively explored for their excellence in molecular designability, facile processability, and low cost, for versatile optoelectronic applications, such as organic light-emitting diodes [1], organic lasers [2], and organic light-emitting transistors [3]. Based on the emission principles, organic emitters can be classified as fluorescence emitters [4], room temperature phosphorescence (RTP) emitters [5], and thermally activated delayed fluorescence (TADF) emitters [6]. Tuning the emission color of organic emitters is essential for optoelectronic applications. Traditional organic emitters with various tunable emission colors are usually achieved through molecular structure design [7,8], bandgap engineering refinement [9–11], and the construction of energy transfer hybrid systems [12–14]. For intelligent optoelectronic applications, i.e., multicolor displays [15,16], visual monitoring [17–19], and information encryption [14,20], it is desirable to develop novel smart responsive organic emitters that are able to achieve facile real-time in situ color tunability in response to external stimuli variations.

Smart responsive organic emitters stand out for their ability to achieve color-tunable emission in response to one or multiple stimuli, including excitation wavelength [21–24], external force [25–28], ultraviolet (UV) illumination [29–32], and delay time [33], corresponding to single-stage and multistage stimuli-responsiveness, respectively. Apparently, multistage stimuli-responsiveness empowers color-tunable emitters with enriched photophysical properties to be modulated by multiple external stimuli, which, in turn, can serve as a valuable platform to investigate the intrinsic photophysical characteristics. As an example, Li et al. [34,35] developed a kind of multicomponent color-tunable emitter with multistage stimuli-responsiveness based on the composite systems consisting of arylboronic acids, dyes, and poly(vinyl alcohol) matrices, exhibiting water/heat-sensitive emission. Wang et al. [36] reported a novel set of multicomponent color-tunable emitters with multistage stimuli-responsiveness whose emission color could be facilely modulated by varying the doping concentration, delay time, and excitation wavelengths. The existing color-tunable emitters with multistage stimuli-responsiveness are generally constructed by integrating multiple components with specific responsive functions [37–41]. In contrast, single-component emitters can avoid cumbersome component integration and overcome the phase separation of the multicomponent systems. Meanwhile, the development of simple single-component color-tunable emitters with multistage stimuli-responsiveness can tackle the limitation issues of smart materials in practical applications. However, it still presents substantial challenges, as most single-component emitters generally have only one single emissive mode, making it rather difficult to achieve multiple response functions to a variety of stimuli.

For the purpose of obtaining color-tunable single-component emitters with multistage stimuli-responsive features, it is a prerequisite to construct multiple emission modes with high sensitivity to different external stimuli in a single-component system. Relevant research in this direction by far has been limited to the attempts to regulate dual-mode emissions [42,43] in single-component systems (Table S1). However, most emitters presented single stimuli-responsive modes. Recently, Song et al. [44] recently reported a type of color-tunable single-component emitters with multistage stimuli-responsiveness through regulating the blue and yellow RTP dual-mode emissions. Nevertheless, the color-tunable range is confined and dual-mode emissions can hardly be modulated to be responsive to a wider range of stimuli. Color-tunable emitters with simultaneous multistage stimuli-responsiveness and multimode emissions in a single-component system are desirable for multifunctional intelligent applications, yet remain challenging.

**Fig. 1.**
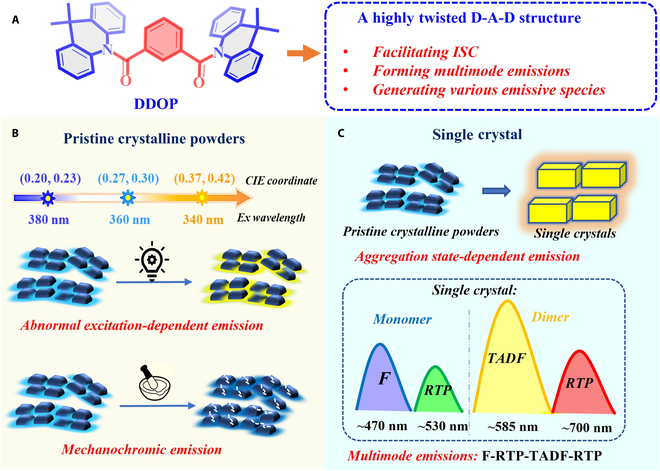
Molecular structure design of the single-component color-tunable emitter DDOP, and its multistage stimuli-responsive emission characteristics. (A) Molecular structure design of DDOP. (B) Emission characteristics of DDOP pristine crystalline powders: excitation-dependent emission and mechanochromic emission. (C) Emission characteristics of the DDOP single crystal: aggregation state-dependent emission and multimode emissions (ISC, intersystem crossing; F, fluorescence; TADF, thermally activated delayed fluorescence; RTP, room temperature phosphorescence).

In this contribution, an interesting example of a single-component color-tunable smart organic emitter, namely, DDOP, is presented, simultaneously demonstrating multistage stimuli-responsive features and multimode emissions. DDOP is distinguished by its highly twisted amide-bridged donor–acceptor–donor (D–A–D) based structure, which has been found to facilitate intersystem crossing (ISC), form multimode emissions, and generate various distinct emissive species responsive to multiple external stimuli in crystals (Fig. 1A). In pristine crystalline powders, it shows excitation-dependent and mechanochromic emissions (Fig. 1B). Meanwhile, it presents a wide emission band with multimode emissions consisting of fluorescence, RTP, TADF, and RTP in single-crystal states. The photophysical characteristics of the pristine crystalline powders are distinctively different from those of single crystals, showing distinctive characteristics closely related to the aggregated states (Fig. 1C). The formation of multiple emission modes in crystals has been further confirmed by single-crystal analysis and theoretical investigations. It is worthy to note that DDOP pristine crystalline powders show abnormal excitation-dependent emission from monomer-dominated blue emission to dimer-dominated yellow emission with the decrease of UV excitation wavelength. Furthermore, inspired by the color-tunable luminescent behaviors, the promising utilizations in multilevel information encryption, multicolor patterns, visual monitoring of UV color, and indirect color-tunable light-emitting diodes (LEDs) have been systematically investigated.

## Results and Discussion

The DDOP molecule is constructed by attaching 9,10-dihydro-9,9-dimethylacridine (DMAC) groups as donor units to a central acceptor isophthaloyl (iPhO) core to afford a twisted D–A–D architecture, which has been found with the following advantages: (a) the D–A–D structure can typically facilitate charge transfer and promote ISC for producing triplet excitons; (b) the twisted D–A structure is also favorable for producing multimode emissions from locally excited (LE) and charge-transfer (CT) excited states; and (c) multiple emissive species can be generated in aggregation states.

The photophysical properties of DDOP were explored in solutions, pristine crystalline powders, and single crystals in details. For dilute solution states, two absorption peaks were recorded at around 245 and 278 nm with a shoulder at nearly 330 nm in dilute tetrahydrofuran (THF) solution (10^−5^ M) (Fig. S7). As shown in Fig. S8, similar photoluminescence (PL) curves with the main emission peaks at around 405 nm were observed when excited at 360 nm in different dilute solutions, which were slightly affected by the polarity of the solvents. According to Fig. S9, concentration-enhanced emission features were observed for DDOP in THF solutions of 10^−5^ to 10^−2^ M. Excitation spectra of DDOP in various concentrations of THF solutions were depicted in Fig. S10. In the 10^−2^ M THF solution, the emission at 405 nm showed a robust excitation peak around 360 nm, which is greatly different from that in the 10^−5^ M dilute solution, indicating that new emissive species might appear with increasing concentration. As shown in Fig. S11, excitation-dependent emissions were observed in the PL spectra of DDOP in 10^−5^ M THF solutions upon excitation at different wavelengths, verifying the presence of multiple emissive species in dilute solutions. As shown in Fig. S12, by increasing the concentration from 10^−5^ to 10^−1^ M, a red-shifted band at 500 nm appeared, which was attributed to excimer fluorescence caused by molecular aggregation.

**Fig. 2.**
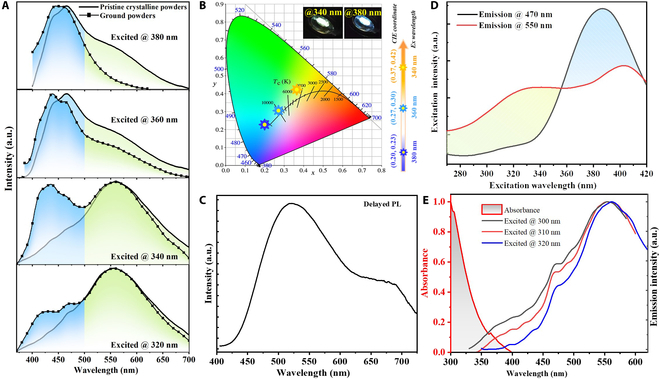
Photophysical characteristics of DDOP pristine crystalline powders. (A) Normalized PL spectra under different excitation wavelengths. (B) The calculated CIE coordinates under different excitation wavelengths (inset: luminescent images when excited at 340 and 380 nm). (C) Delayed PL spectra at room temperature. (D) Excitation spectra of different emission wavelengths. (E) Normalized absorption and PL spectra excited at 320 nm.

As for pristine crystalline powders, the emission color was modulated from yellow to blue via increasing the excitation wavelength, exhibiting an attractive excitation-dependent emission. As shown in Fig. 2A, the emission curves excited at 320 and 340 nm exhibited yellow-dominated emission peaking at 550 nm with small shoulders at around 470 nm with the photoluminescence quantum yield (PLQY) of 5.8% (excited by 320 nm). In contrast, the main emission bands were located in a blue region accompanied with shoulder peaks in a yellow region when excited at 360 nm or 380 nm with the PLQY of 8.3% (excited by 380 nm). A nearly linear variation of Commission International de l’Eclairage (CIE) coordinates from (0.37, 0.42) to (0.27, 0.30) and then to (0.20, 0.23) was observed accordingly when the excitation wavelength was increased from 340 nm to 360 nm, and subsequently to 380 nm (Fig. 2B). According to the delayed PL spectra in Fig. 2C, it was observed that pristine crystalline powders presented a main emission peak at 520 nm with a shoulder peak at around 700 nm at room temperature. As shown in Fig. 2D, the yellow emission centered at 550 nm was more intense than the blue emission centered at 470 nm when excited by the UV wavelength from 270 to 355 nm, whereas blue-dominated emission was observed with the excitation in the range from 355 to 420 nm. The change of proportions of the emissions centered at 470 and 550 nm in the case of various excitation wavelengths could lead to the color-tunable emission. To further clarify the possible reasons for the abnormal excitation-dependent emission, the PL measurement range of DDOP has been expanded to the UV region. As shown in Fig. 2E, when pristine crystalline powders were excited at the shorter UV wavelengths, such as 300 nm, 310 nm, and 320 nm, there were emission signals in the UV-visible region (from 350 to 400 nm), which corresponded to the emission of DMAC groups and was thought to originate from the LE state [45]. It is worthwhile to mention that one excitation band of 550 nm is in accordance with the LE emission of DDOP, confirming that the LE emission of DDOP can excite the emission of 550 nm. Another excitation band of 550 nm at the shorter UV wavelength (from 270 nm to 355nm) can also excite the LE emission. It can be inferred that the shorter UV wavelengths, such as 320 nm and 340 nm, can excite the emission of 550 nm through radiative energy transfer from LE emission to the emission of 550 nm [46]. Meanwhile, there were absorption signals in the region from 300 to 400 nm, which had a certain spectral overlap with the emission from LE state (Fig. 2E), further confirming the presence of energy transfer [47]. A comparison of Fig. 2D and E demonstrated the obvious differences between the excitation and absorption spectra, which further indicated the existence of multiple emissive species in DDOP [48].

The yellow emission at 550 nm of pristine crystalline powders originated from the new emissive species of the molecular dimers in crystalline aggregates. To clear this up, DDOP was dispersed in poly(methyl methacrylate) (PMMA) matrices (5 wt. %) to abolish the influence of intermolecular interactions [49]. As depicted in Fig. S13, only a fluorescence peak of 470 nm was recorded, where the intermolecular interactions were weakened and the emission was ascribed to the monomer. Hence, the emission at 470 nm for the pristine crystalline powders was supposed to be a monomer-dominated emission, whereas the emission at 550 nm came from molecular dimers in the aggregates. As shown in Fig. S14 and S15 and Table S3, the lifetimes of two emission peaks of 470 nm and 550 nm were both nanosecond (ns) levels based on the decay profiles of DDOP pristine crystalline powders. The average lifetime of the delayed emission at 520 nm was 501.89 μs (Fig. S16 and Table S3). With various excitation wavelengths at low temperatures in a liquid nitrogen environment, the PL spectra of pristine crystalline powders presented a main emission peak at nearly 470 nm, but the emission peak at 550 nm was hardly detected (Fig. S17), which might be caused by the suppressed energy transfer due to the hindered collision of molecules under low temperature. In addition, the formation of the molecular dimer for pristine crystalline powders might be suppressed due to the restricted molecular motion at low temperature. Thus, the emission of 550 nm originating from the dimer in crystalline aggregates might be suppressed [50].

Inspiringly, DDOP demonstrated mechanochromic emission features. The ground powders also presented color-tunable emission under different excitation wavelengths (Fig. 2A and Fig. S18). After grinding, the emission at about 470 nm was slightly blue-shifted, together with the increasing proportion of blue emission components. In particular, under excitation at 340 nm, almost white emission was observed with equally ratiometric blue and yellow emissions from the grinding powder, manifesting an obvious difference from the pristine crystalline powders. Such mechanochromic behaviors resulted from the destruction of crystallinity under external mechanical stimuli, which was corroborated by the weakened powder x-ray diffraction patterns (Fig. S19).

**Fig. 3.**
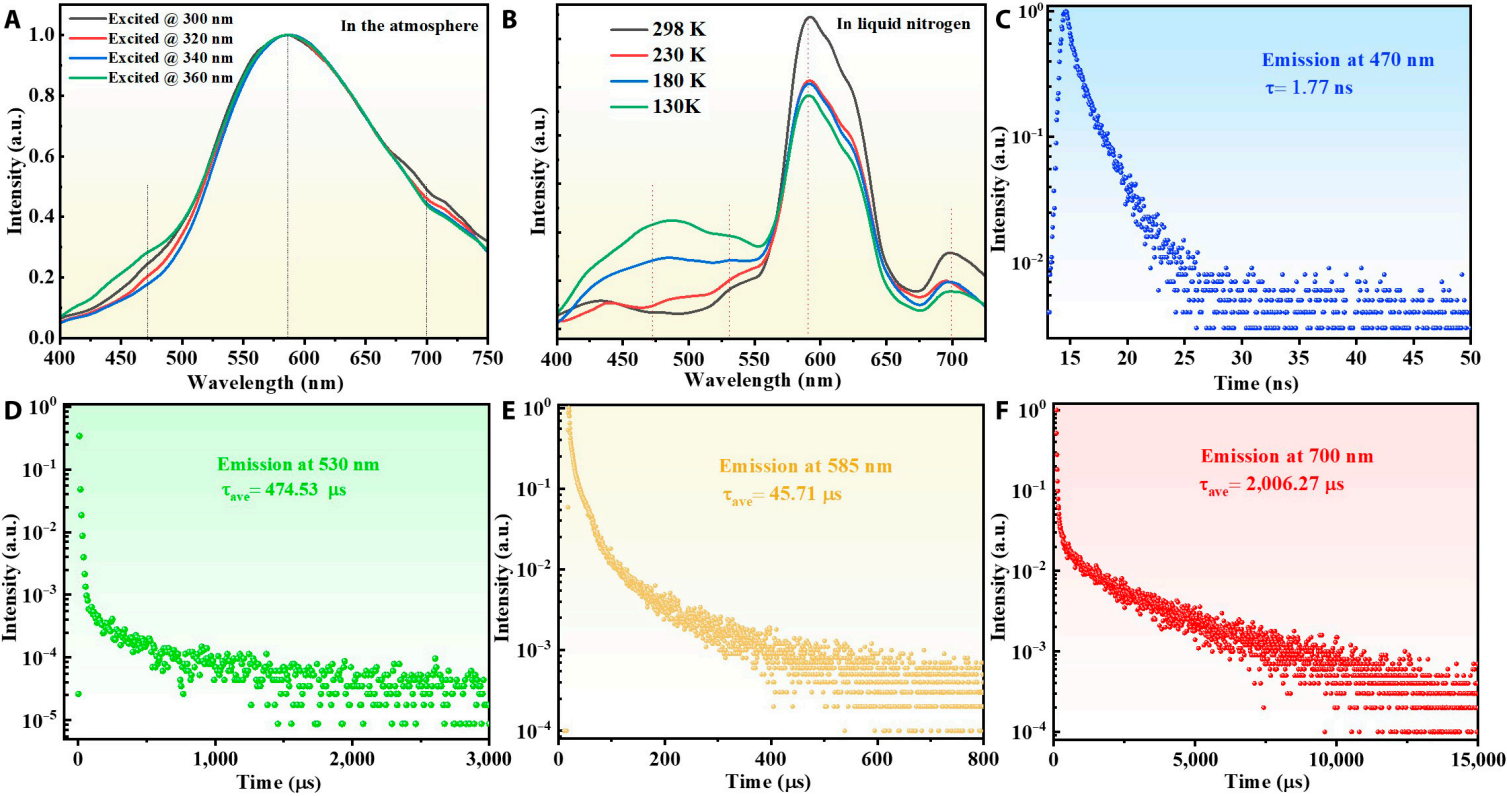
Photophysical properties of DDOP single crystals. (A) Normalized PL spectra excited by different wavelengths in the atmosphere. (B) The variable temperature emission spectra excited at 360 nm in liquid nitrogen. Decay curves of different emission peaks at (C) 470 nm, (D) 530 nm, (E) 585 nm, and (F) 700 nm.

Meanwhile, photophysical properties of DDOP single crystals were systematically investigated. As shown in Fig. 3A, broad emission bands centered at 585 nm could be detected under different excitation wavelengths with the PLQY of 9.8% (excited by 380 nm), and two shoulder peaks located at around 470 nm and 700 nm. Meanwhile, weak LE emissions of 400 nm were also recorded in single-crystal states. According to the delayed PL spectra in Fig. S20, DDOP single crystals exhibited two delayed emission bands peaking at around 530 nm and 700 nm, respectively. The two delayed emission peaks of 530 nm and 700 nm in single crystals were almost consistent with the two delayed emission peaks of pristine crystalline powders. By comparing with the PL curve of DDOP dispersed in PMMA (Fig. S13), it can be confirmed that the emission at 470 nm stemmed from the monomer, and the emission at 585 nm and 700 nm stemmed from dimers in aggregate states. Considering the emission peaks emerging in PL and delayed PL spectra, it could be concluded that the wide emission band of DDOP single crystals consisted of multimode emissions centered at 470 nm, 530 nm, 585 nm, and 700 nm, respectively. DDOP single crystals presented dimer-dominated emissions centered at 585 nm, which was red-shifted in comparison with the dimeric emission of pristine crystalline powders (Fig. S21). The red-shifted emission should be attributed to the tight and robust intermolecular interactions for single crystals. In a word, the photophysical properties of DDOP single crystals were different from those of pristine crystalline powders, demonstrating aggregation state-dependent features.

To identify the type of these multiple emission peaks, the variable temperature emission spectra in liquid nitrogen, lifetime decay profiles, and delayed PL spectra at low temperature were recorded in Fig. 3B, Fig. 3C to F, and Fig. S22, respectively. As shown in Fig. 3B, the finer emissions with more obvious multimode emission peaks were observed in a nitrogen environment. According to Fig. S22, the emission bands of 530 nm and 700 nm were observed at low temperature. The emission peak at 470 nm had typical fluorescence characteristics with an average lifetime of 1.77 ns (Fig. 3C). The intensity of the emission peak at 530 nm was increased by decreasing temperature (Fig. 3B), and its average lifetime was up to 474.53 μs, featuring RTP emission (Fig. 3D). The emission intensities at around 585 nm were enhanced with the increment of temperature from 130 K to 298 K (Fig. 3B), and it can be attributed to TADF emission combined with the average excited-state lifetime around 45.71 μs at room temperature (Fig. 3E). The emission intensities of red emission at 700 nm do not exactly follow a common decreasing trend with increasing temperature, but its RTP properties can be explained on the basis of the emerged delay PL at low temperature (Fig. 3B) and an average excited-state lifetime of about 2,006.27 μs at room temperature (Fig. 3F). As for the RTP emission at 700 nm, it originated from the dimeric phosphorescence, which can be affected by both temperature and molecular motions [50]. The dimeric emission played a dominant role at room temperature due to proper molecular motions to generate robust interactions in dimers, resulting in a strong emission intensity at 700 nm with the temperature approaching room temperature as shown in Fig. 3B, which could explain the abnormal trend of variations with increasing temperature. Furthermore, PL spectra of isolated molecules in dilute 2-methyltetrahydrofuran solution at low temperature in liquid nitrogen were also studied, whose phosphorescence band located at a blue region consisting of two peaks at 405 and 428 nm (Fig. S27). The single crystals exhibited the phosphorescence at 530 nm and 700 nm, originating from crystalline aggregates rather than isolated molecules. To sum up, the DDOP single crystals featured distinct fluorescence–RTP–TADF–RTP multimode emissions with peaks located at 470 nm, 530 nm, 585 nm, and 700 nm, respectively, which could be closely associated with the effective intermolecular interactions and tightly packing modes in single crystals.

**Fig. 4.**
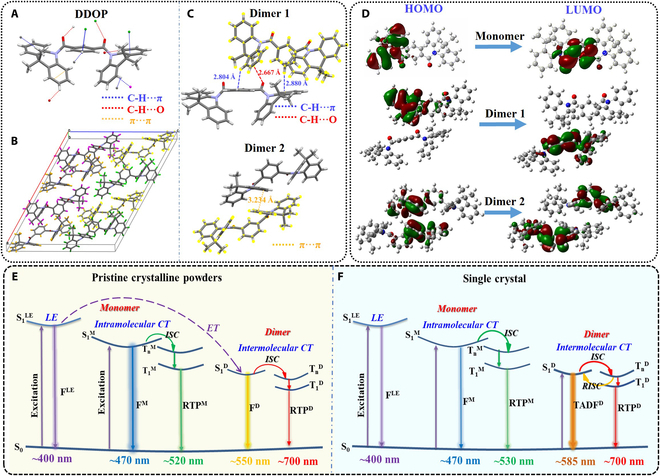
Single crystal, theoretical calculations, and emission-mechanism analyses of DDOP. Intermolecular interactions of (A) monomer, (B) molecular packing, and (C) dimers 1 and 2 in the DDOP single crystal. (D) Frontier molecular orbital distributions for the selected monomer and dimers 1 and 2 in the DDOP single crystal. Emissive mechanism insights of DDOP pristine crystalline powders (E) and single crystals (F). (LE, locally excited; CT, charge transfer; ET, energy transfer; ISC, intersystem crossing; RISC, reverse intersystem crossing; F, fluorescence; TADF, thermally activated delayed fluorescence; RTP, room temperature phosphorescence; M, monomer; D, dimer.)

In order to explore how molecular stacking and intermolecular interactions affect the emission characteristics, crystallographic analysis was conducted. Based on the single-crystal structure in Fig. 4A, the DDOP molecule presented a highly twisted conformation with proper torsion angles between DMAC donors and acceptors. As indicated in Fig. 4B, eight twisted DDOP molecules were included in each cell unit, and every molecule was closely interacted with neighbor molecules through robust intermolecular interactions (C-H∙∙∙π, C-H∙∙∙O, etc.). According to Fig. 4C, adjacent molecules were interlocked by C-H∙∙∙π and C-H∙∙∙O interactions between DMAC and iPhO units in dimer 1, and effective π–π interactions existed between two DMAC units in dimer 2. In addition, C-H∙∙∙π intermolecular interactions were generated in dimer 3 (Fig. S28). Moreover, the effective C-H∙∙∙O intermolecular interactions between DMAC groups as electron donors and ketone moieties as electron acceptors in dimer 1 can result in the formation of intermolecular CT states [33,51]. The intermolecular interactions in single crystals were further calculated and analyzed via the independent gradient model (IGM) (Figs. S29 and S30). The large IGM isosurfaces were produced between adjacent molecules in dimer 1 and dimer 2 (Fig. S30), demonstrating that there were robust intermolecular interactions in the dimers. All of these effective intermolecular interactions could inhibit molecular motions to weaken nonradiative relaxation, which is beneficial for generating TADF and RTP emissions. Besides, the tight molecular stacking and robust intermolecular interactions in single-crystal states were responsible for the red-shifted TADF emission of 585 nm for DDOP single crystals. As shown in Raman spectra (Fig. S31), the Raman signals of C=O vibration in the DDOP single crystal showed obvious shifts compared to those of the isophthalic acid fragment, confirming the existence of the robust C-H∙∙∙O intermolecular interactions [52], whereas DDOP pristine crystalline powders were hardly detected in Raman signals. As indicated, the intermolecular interactions in single crystals were stronger than those in powders, which could suppress the nonradiative transition of DDOP molecules in a more effective way.

Theoretical calculations were carried out to further explore the intrinsic emission mechanism. The models of monomer and selected dimers were determined based on the single crystal. As shown in Fig. 4D, the highest occupied molecular orbital (HOMO) and the lowest unoccupied molecular orbital (LUMO) were separately located on DMAC donor and iPhO acceptor units in the monomer, verifying intramolecular charge transfer. For dimer 1 and dimer 2, the HOMO and LUMO were located in different molecules for generating intermolecular charge transfer. The nature transition orbital analysis further revealed that both monomer and dimers produced CT-dominated excited states (Figs. S32 and S33). For the monomer, there was one main transition channel from S_1_ to T_1_ for the ISC (Fig. S34), whereas the number of ISC channels (from S_1_ to T_7_, T_8_, T_9_, and T_10_) was notably increased for dimer 1 (Fig. S35). In the case of dimer 1, the calculated energy gap between the involved singlet excited state (S_1_) and triplet excited states (T_10_) was 0.26 eV (<0.37 eV), which further demonstrated the possibility for the coexistence of RTP and TADF in this single-crystal state.

The proposed emission mechanisms of DDOP are summarized in Fig. 4E and F. Together with photophysical properties, both DDOP pristine crystalline powders and single crystals generated multimode emissions from LE–monomer–dimer multiple emissive species. Theoretical calculations demonstrated that intramolecular and intermolecular charge transfer emerged from monomers and dimers, respectively. Meanwhile, there were effective ISC channels for promoting the generation of triplet excitons in both monomers and dimers. For DDOP pristine crystalline powders (Fig. 4E), the emissions of fluorescence centered at 470 nm and RTP centered at 520 nm were related to intramolecular charge transfer in the monomer, and the emissions of fluorescence centered at 550 nm and weak RTP centered at 700 nm stemmed from intermolecular charge transfer in the dimer. Furthermore, the longer UV wavelengths (360 nm and 380 nm) could directly excite the monomer-dominated emission centered at 470 nm, while the shorter UV wavelengths (320 nm and 340 nm) could excite the dimer-dominated emission centered at 550 nm via the radiative energy transfer from the LE state to the dimeric state. Consequently, DDOP pristine crystalline powders presented abnormal excitation-dependent emission features. In contrast, DDOP single crystals produced a wide emission band consisting of fluorescence, RTP, TADF, and RTP, with a main emission peak at 585 nm. As demonstrated in Fig. 4F, the monomeric emissions of the single crystals included fluorescence of 470 nm and RTP of 530 nm, which was almost consistent with pristine crystalline powders. Meanwhile, the dimeric emissions of the single crystals included TADF centered at 585 nm and RTP centered at 700 nm, which were associated with intermolecular charge transfer. It should be mentioned that the dimer-dominated emission peak at 585 nm in the single crystals was red-shifted in comparison with the dimeric emission of pristine crystalline powders, which was related to the robust intermolecular interactions in DDOP single crystals. Meanwhile, the robust intermolecular interactions in dimers can inhibit the nonradiative transition of triplet excitons, which is also beneficial for generating the TADF and RTP emission in the dimer.

**Fig. 5.**
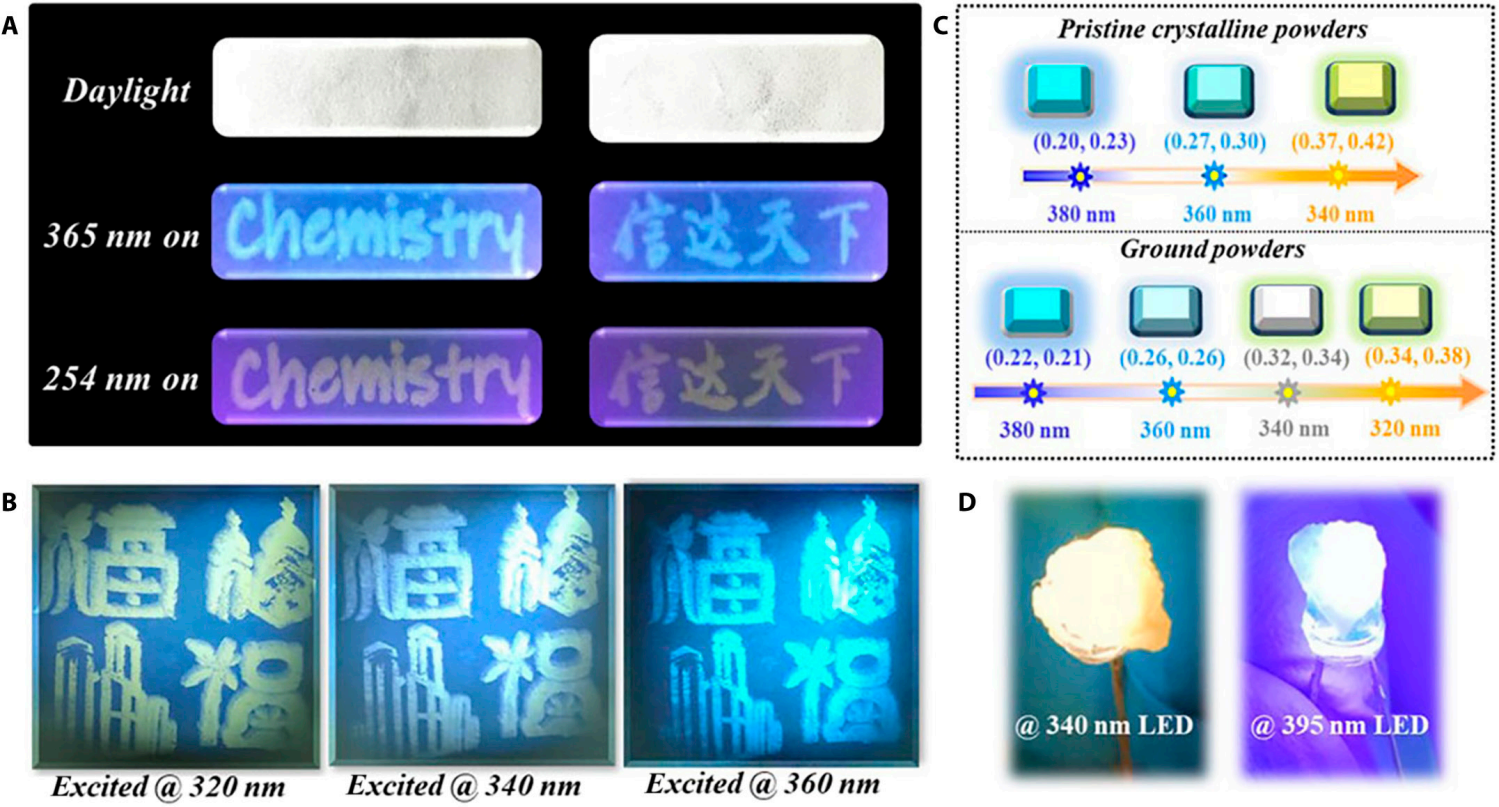
Applications of color-tunable DDOP emitters. (A) Multilevel information encryption using DDOP as the ink. (B) Multicolor display photographs of Chinese word “Fu” patterns fabricated by the ground powders varying the excitation wavelength of 320, 340, and 360 nm. (C) Excitation-dependent emissive color chart with the corresponding CIE coordinates. (D) Indirect color-tunable LEDs covered by DDOP powders @PDMS elastomers.

Inspired by the excitation-dependent color-tunable emission features, reliable information encryption at much higher levels is expected. To prove this, information encryption experiments were performed. As shown in Fig. 5A, the information loaded on the filter paper in advance was hardly observed on daylight. When excited with various UV lights, the information was observed with different emissive colors. In addition, a multicolor emissive pattern of the Chinese words “Fu” was fabricated via screen printing techniques, whose inks were made by dispersing the ground DDOP powders into commercial aloe vera gels. The emissive color of the pattern was changed from yellow-green to white and then to blue by modulating the excitation wavelength from 320 nm to 340 nm and then to 360 nm (Fig. 5B). The variations of CIE coordinates and excitation wavelengths had almost linear relationship, demonstrating the potential in acting as visual sensing charts for distinguishing UV wavelengths. As shown in Fig. 5C, the varied emission colors and CIE coordinates could reflect the UV excitation wavelengths in both pristine crystalline powders and ground powders. As presented in Fig. 5D, indirect color-tunable LEDs from yellow to blue emission were fabricated by encapsulating DDOP pristine crystalline powders in PDMS elastomers, and then attaching the DDOP powders @PDMS elastomers to LEDs with different UV emission wavelengths of 340 nm and 395 nm.

## Conclusion

In summary, we have developed a single-component color-tunable smart organic emitter (DDOP) with multistage stimuli-responsiveness based on a highly twisted amide-bridged D–A–D structure, which facilitates the generation of a variety of distinct emissive species that are responsive to multiple external stimuli. DDOP pristine crystalline powders exhibit color-tunable emissions from yellow green to blue by increasing the UV excitation wavelength and demonstrate blue-shifted mechanochromic emission under anisotropic grinding, whereas the single crystals produce a wide emission band consisting of fluorescence, RTP, TADF, and RTP, with a main emission peak at 585 nm. The emission behaviors of pristine crystalline powders and single crystals are different, exhibiting aggregation state-dependent emission features. According to the results of experiments and calculations, it can be proved that DDOP has produced multimode emissions from LE–monomer–dimer multiple emissive species, which can be regulated by multiple external stimuli for producing color-tunable emissions with multistage stimuli-responsiveness. This study not only develops color-tunable single-component smart organic emitters with simultaneous multistage stimuli-responsiveness and multimode emissions but also reveals intrinsic emissive mechanisms in the process, which is vital for expanding intelligent optoelectronic applications, including multilevel information encryption, multicolor emissive patterns, and visual monitoring of UV wavelengths*.*

## Materials and Methods

### Material synthesis

The starting materials, such as 9,10-dihydro-9,9-dimethylacridine, isophthaloyl chloride, and potassium carbonate, were commercially available, which were used as received. All chemicals were purchased from commercial suppliers and used directly without further purification. The target products were synthesized according to the following methods: 9,10-dihydro-9,9-dimethylacridine (1.05 g, 5 mmol) and potassium carbonate (1.38 g, 10 mmol) were added into a 100-ml flask and dissolved in toluene (20 ml) and stirred for about 20 min at 100°C. Then, isophthaloyl chloride (1.02 g, 5 mmol) was dropped in the above resultant mixture and stirred overnight. The reaction process was monitored by thin-layer chromatography. The final resultant mixture was extracted with dichloromethane and saturated sodium chloride solution. The combined organic extracts were dried over anhydrous Na_2_SO_4_ and concentrated by rotary evaporation. The crude product was purified by column chromatography on silica gel using petroleum ether and dichloromethane (1:2, V: V) as eluent, and 2 times recrystallization from dichloromethane and *n*-hexane to afford yellow pristine crystalline powders (target product) in a yield of 45%.

### Crystal cultivation

Single crystals of DDOP were cultivated through dissolving the product (150 mg) into a solvent mixture containing 6 ml of dichloromethane and 3 ml of methanol. Then, the solution was kept under ambient conditions to let the solvent evaporate slowly. Yellow transparent block-like crystals were formed in the solution.

### Measurements

Nuclear magnetic resonance (^1^H and ^13^C NMR) spectra were collected using Bruker ARX400 Nuclear Magnetic Resonance. The chemical shift was relative to tetramethylsilane as the internal standard. Resonance patterns were reported with the notation of s (singlet), d (doublet), t (triplet), q (quartet), and m (multiplet). High-resolution mass spectrum was measured on Waters G2-XS QTOF in a positive ion mode. High-performance liquid chromatogram spectrum was recorded on Agilent 1290II. UV–Visible absorption spectra were obtained using Perkin Elmer Lambda 35. Steady-state PL spectra and excitation spectra were measured using Hitachi F-4600. The delayed PL spectra and lifetimes were measured on an Edinburgh FLSP 980 spectrophotometer equipped with a xenon arc lamp (Xe 900) and microsecond flash-lamp (μF900), respectively. The PLQYs were measured on an Edinburgh FLSP 980 spectrophotometer equipped with an integrating sphere. The variable temperature emission spectra were measured on OLYMPUS BX53M with the constant excitation wavelength of 360 nm under nitrogen atmosphere. Single-crystal x-ray diffraction experiments were carried out using a Bruker D8 QUEST x-ray single crystal diffractometer. Luminous patterns of information encryption and display under different UV excitation wavelengths were taken by cell phone in professional mode.

### Computational details

The analysis of the IGM was carried out by Multiwfn 3.6 [53,54] and was volume rendered by VMD 1.9.3 based on the DDOP crystal data in initial state.

Density functional theory (DFT) and time-dependent DFT (TD-DFT) simulations were performed with Gaussian 16 package [55]. All the computational models were built from the single-crystal structures without further geometry optimization.

The excitation energy of the nth singlet state (S_n_) and the nth triplet state (T_n_) was calculated using the TD-DFT method of B3LYP/6-31G(d) level based on the monomer and selected dimers extracted from the single crystal. Frontier molecular orbital distributions were calculated based on the monomer and selected dimers extracted from the single-crystal at B3LYP/6-31G(d).

## Data Availability

All data that support the findings of this study are available from the corresponding author upon reasonable request.

## Supplementary Materials

Supplementary 1 Tables S1 to S8 Scheme S1 Figs. S1 to S35
