# Supplementary material for: Single-Component Color-Tunable Smart Organic Emitters with Simultaneous Multistage Stimuli-Responsiveness and Multimode Emissions
Source: Research (Wash D C). 2023 Sep 28;6:0241. doi: 10.34133/research.0241 (PMC10539023; doi:10.34133/research.0241)
Supplement: Supplementary 1 — Tables S1 to S8 Scheme S1 Figs. S1 to S35 [file research.0241.f1.pdf]

Supporting Information

Single-Component Color-Tunable Smart Organic Emitters with Simultaneous Multistage Stimuli-Responsiveness and Multimode Emissions

Yu Yan[1], Chengfang Liu[1], Jianzhong Fan[2], Yusheng Li[1], Huanling Liu[2], Qian Wang[1], Xiangchun Li[1], Junfeng Li[1], Wen-Yong Lai[1]*

[1]State Key Laboratory of Organic Electronics and Information Displays (SKLOEID)

Institute of Advanced Materials (IAM), School of Chemistry and Life Sciences

Nanjing University of Posts & Telecommunications

9 Wenyuan Road, Nanjing 210023, China

[2]Shandong Province Key Laboratory of Medical Physics and Image Processing Technology

School of Physics and Electronics

Shandong Normal University

Jinan, 250358, China

*Address correspondence to: iamwylai@njupt.edu.cn

Table S1. Stimuli-responsive emissions from color-tunable smart organic emitter DDOP compared to those recently reported.

Compound	Stimulus type	Emission mode	Color-tunable range	References
1	Mechanical force	F: Monomer, excimer	Purple to blue<br>PyBpin: 403 nm to 466 nm<br>PPCHO: 423 nm to 461 nm	*Adv. Opt. Mater.* 2018, 6, 1800198.<br>*Adv. Opt. Mater.* 2020, 8, 1902036.
2	Self-assembly	F: Monomer, aggregates	Blue to yellow<br>420 nm to 530 nm	*Nat. Commun.* 2020, 11, 158.
3	Excitation wavelength	F: Keto-form, excimer	Green to yellow<br>526 nm to 593 nm	*Angew. Chem. Int. Ed.* 2019, 58, 8773.
4	Mechanical force	F, RTP	Green to blue<br>497 nm to 426 nm	*Angew. Chem. Int. Ed.* 2017, 56, 15299.
5	1) Mechanical force<br>2) Excitation light	F, DF	1) Fluorescence red-shift：Purple to blue 418 nm to 443 nm<br>2) Reversible crystal-state photochromism.	*Angew. Chem. Int. Ed.* 2019, 58, 16445.
6	Mechanical force	F, DF	Blue to yellow<br>445 nm to 555 nm	*Adv. Opt. Mater.* 2019, 7, 1801667.
7	1) Temperature;<br>2) Excitation intensity;<br>3) Mechanical force.	F, RTP	Blue to yellow<br>424 nm to 547 nm	*Adv. Funct. Mater.* 2021, 31, 2101312.
8	Excitation wavelength	RTP: Monomer, aggregates	Blue to green<br>465 nm to 505 nm	*Nat. Photonics* 2019, 13, 406.
9	Excitation wavelength	RTP: Monomer, clusters	Blue to green<br>470 nm to 530 nm	*Nat. Commun.* 2022, 13, 429.
**DDOP**	1) Excitation wavelength;<br>2) Mechanical force;<br>3) Aggregation state	**F, DF, RTP**	1) **Blue to yellow with decreasing UV excitation wavelength** 470 nm to 550 nm;<br>2) Blueshifted mechanochromic emission<br>3) **Full-color emission** in single crystals	**This work**

(M: Monomer; D: Dimer; F: Fluorescence; TADF: Thermally activated delayed fluorescence; RTP: Room temperature phosphorescence)

The structures of compounds 1-9 are listed as follows:

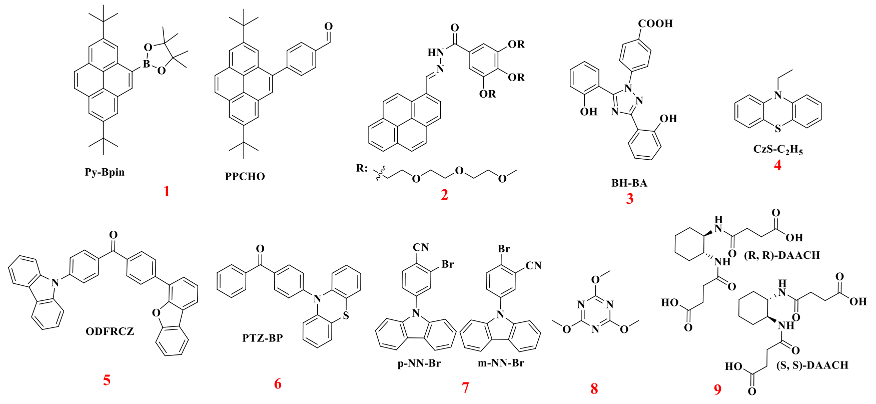

Material synthesis

The target products were synthesized according to Scheme S1. The molecule synthesized was purified by column chromatography and recrystallization from dichloromethane and *n*-hexane for two times. ^{1}H NMR (400 MHz, $CDCl_3$): δ 7.68 (*d*, 1H), 7.47-7.52 (*d*, 4H), 7.38-7.43 (*d*, 4H), 7.30(*d*, 1H), 7.27(*d*, 1H), 7.18-7.25 (*m*, 4H), 7.06-7.17 (*m*, 5H). ^{13}C NMR (100 MHz, $CDCl_3$): δ 166.45, 141.02,138.71, 136.21, 130.13, 129.81, 127.08, 126.03, 125.80, 125.62, 123.56, 38.16, 26.67.

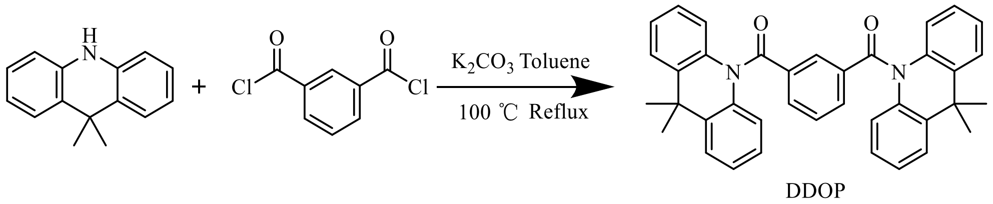

Scheme S1. Synthetic routes of the target compound DDOP.

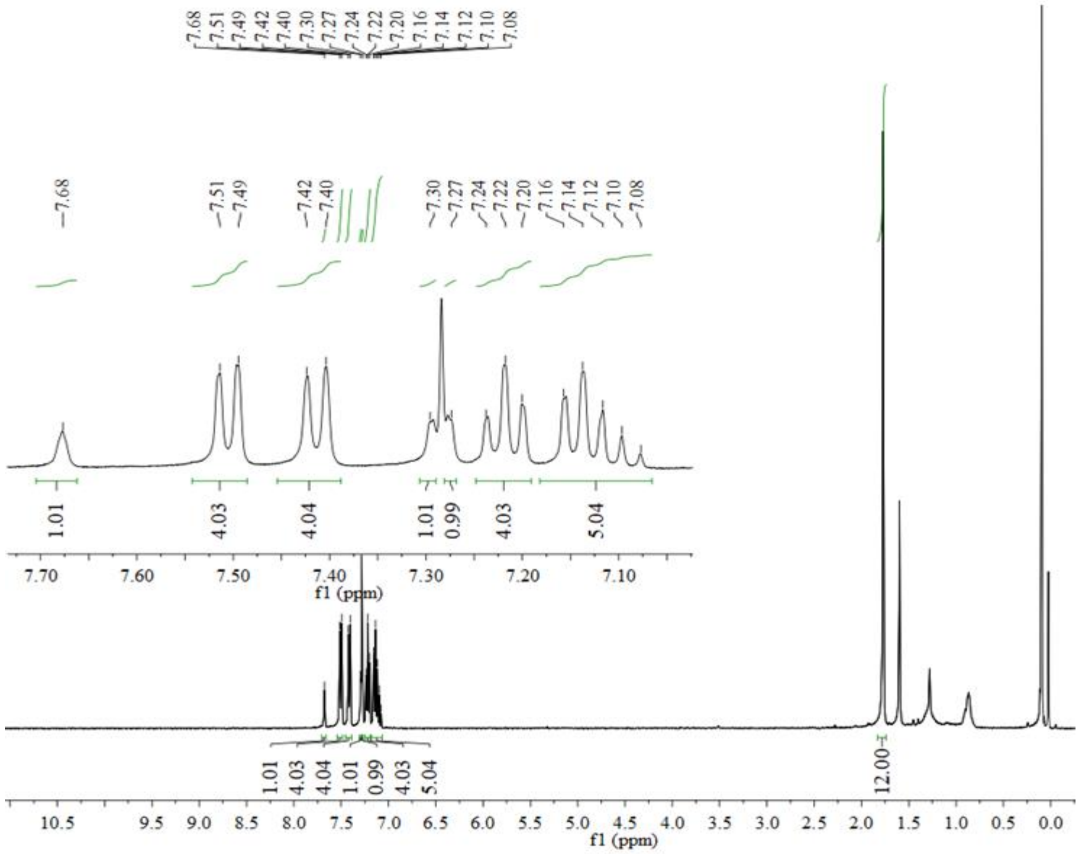

Figure S1. ^{1}H NMR spectra of DDOP in $CDCl_3$ solution.

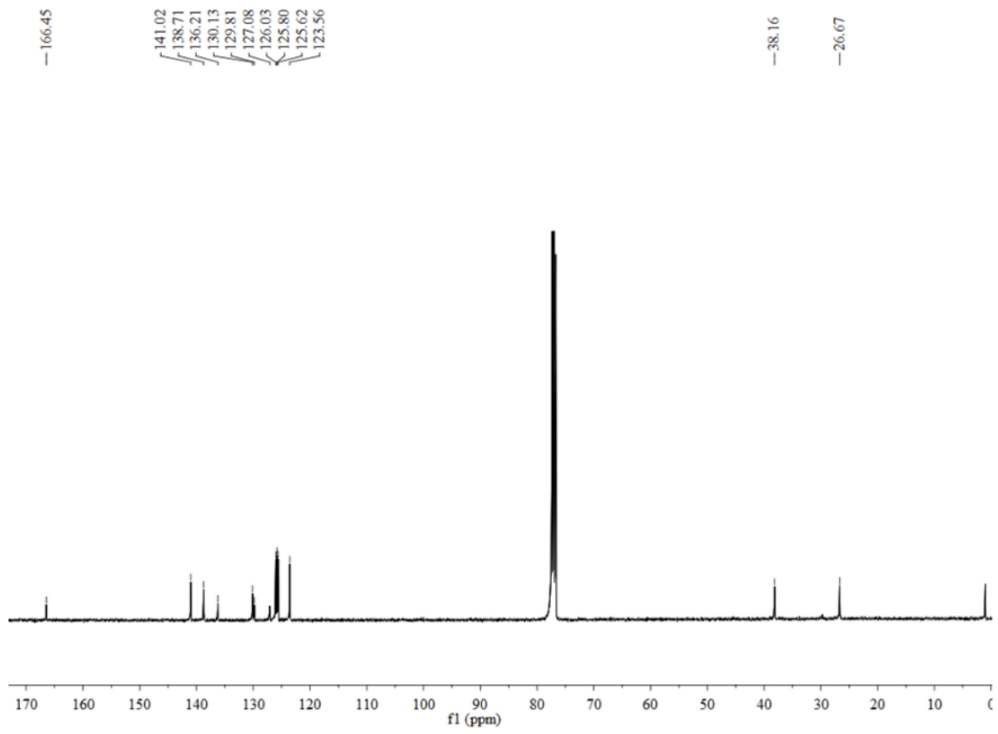

Figure S2. ^{13}C NMR spectra of DDOP in $CDCl_3$ solution.

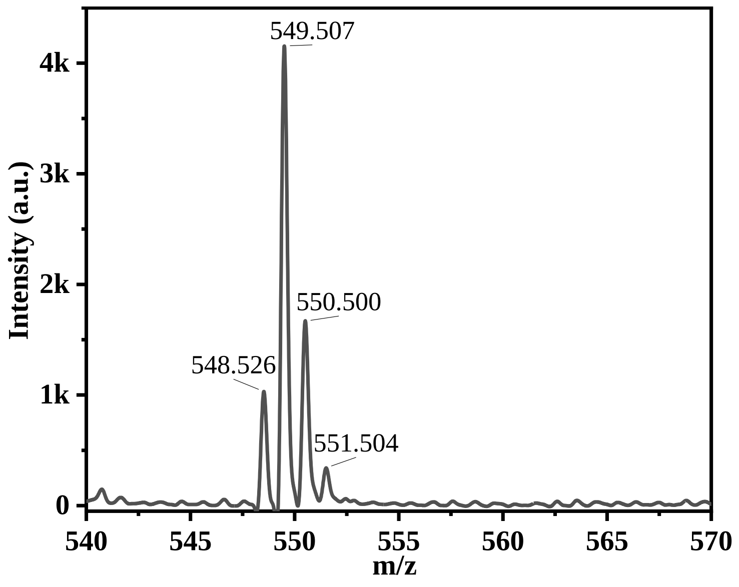

Figure S3. MALDI-TOF spectra of DDOP.

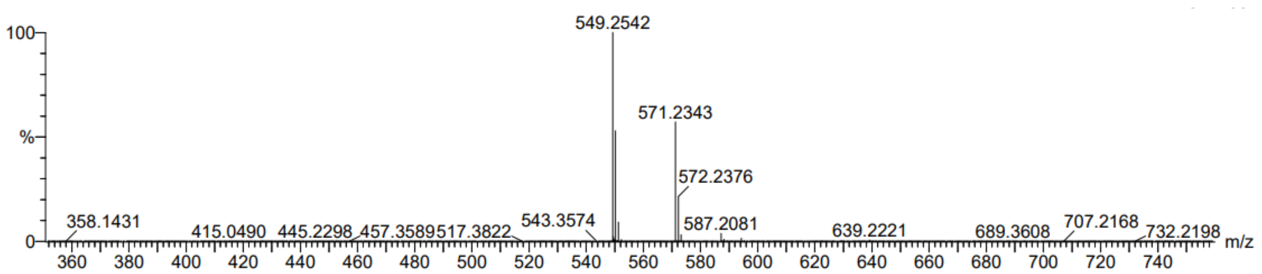

Figure S4. HRMS spectra of DDOP.

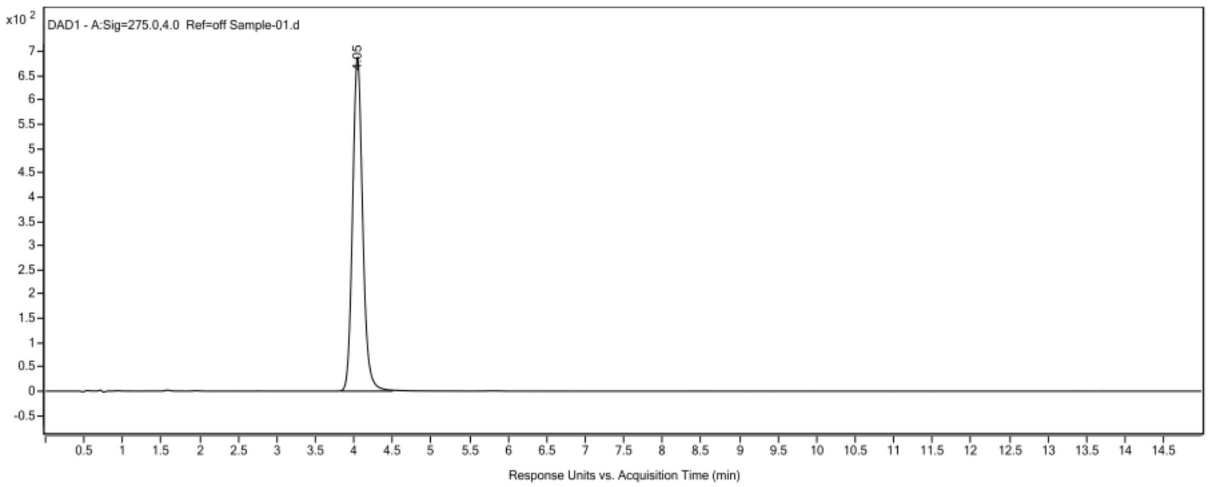

Figure S5. HPLC spectra of DDOP.

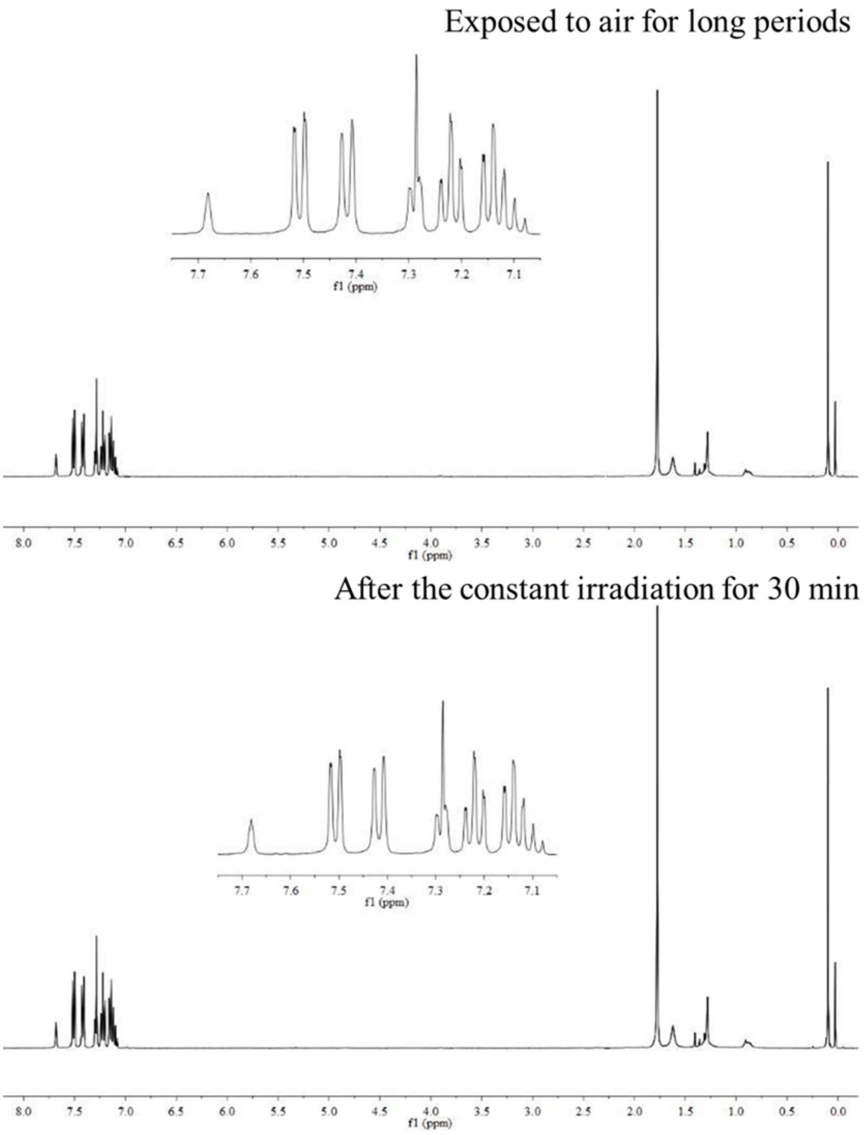

Figure S6. ^{1}H NMR spectra of DDOP in $CDCl_3$ solution under the constant irradiation and exposed to air for long periods of time.

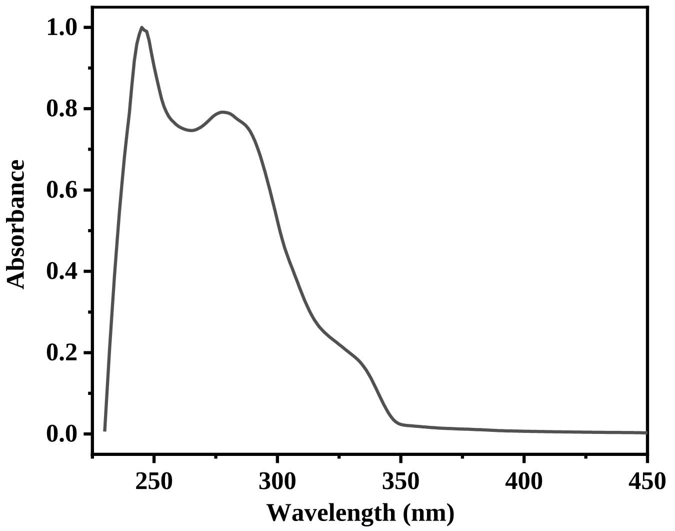

Figure S7. Normalized absorption of DDOP in dilute THF solution (10^{-5} M) under ambient conditions.

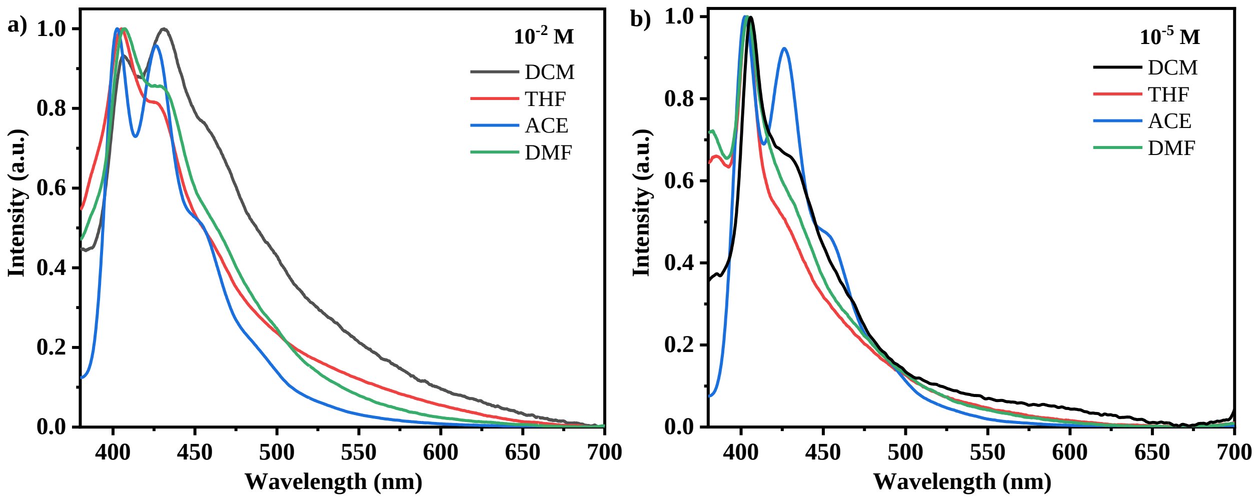

Figure S8. Normalized PL spectra of DDOP excited at 360 nm in different solvents (DCM, THF, ACE and DMF) at room temperature. a) 10^{-2} M; b) 10^{-5} M.

Table S2. Photophysical properties of DDOP excited at 360 nm in different solvents (DCM, THF, ACE and DMF) at room temperature.

Solvent	Emission wavelength (nm)						
	10^{-2} M			10^{-5} M			
DCM	406	431	455		406	431	
THF	405	427	455	385	403	426	
ACE	403	426	452		402	426	454
DMF	407	432	457	383	404	429	

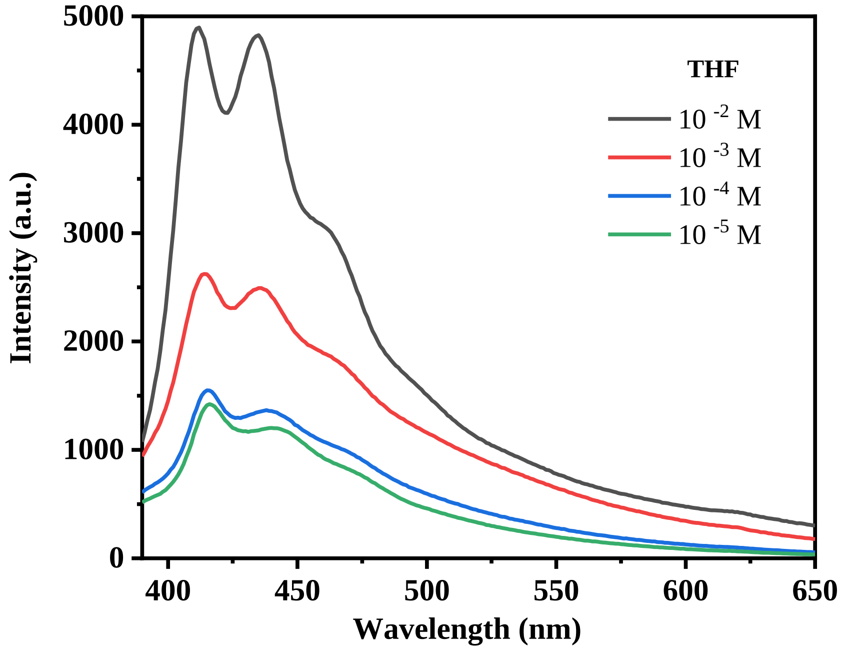

Figure S9. PL spectra of DDOP excited at 360 nm in variable concentrations of THF solutions at room temperature.

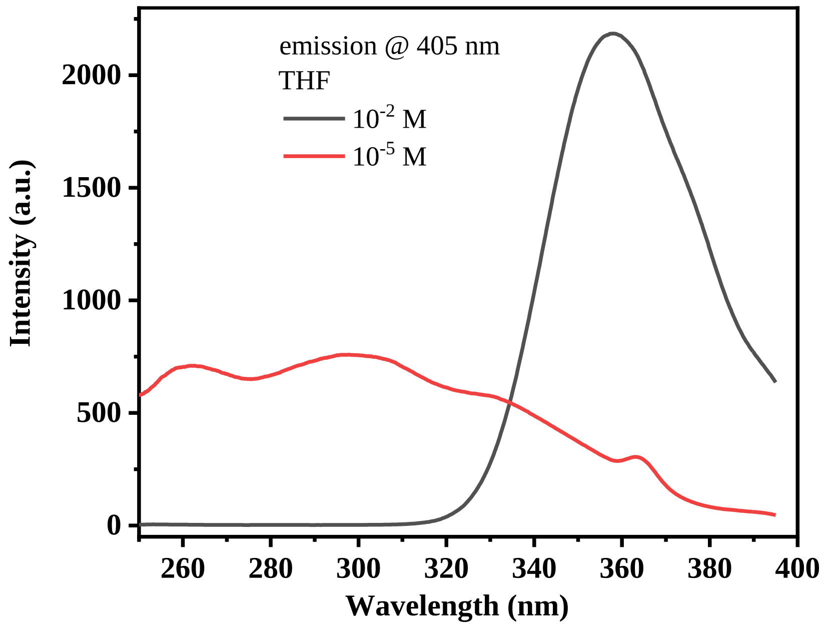

Figure S10. Excitation spectra of DDOP in variable concentrations of THF solutions at room temperature.

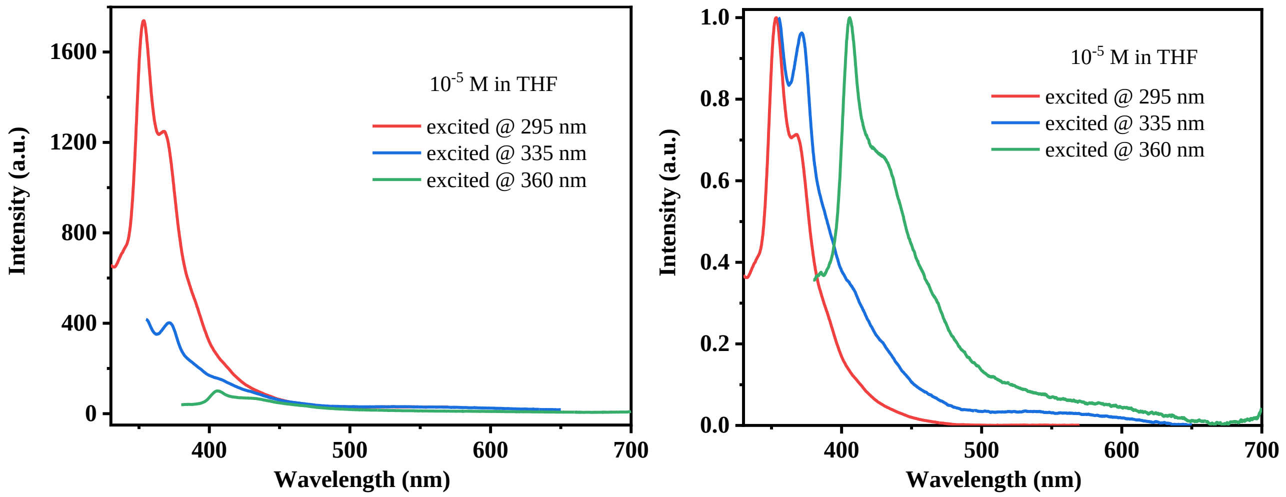

Figure S11. PL spectra of DDOP in 10^{-5} M THF solution excited at different excitation wavelengths at room temperature.

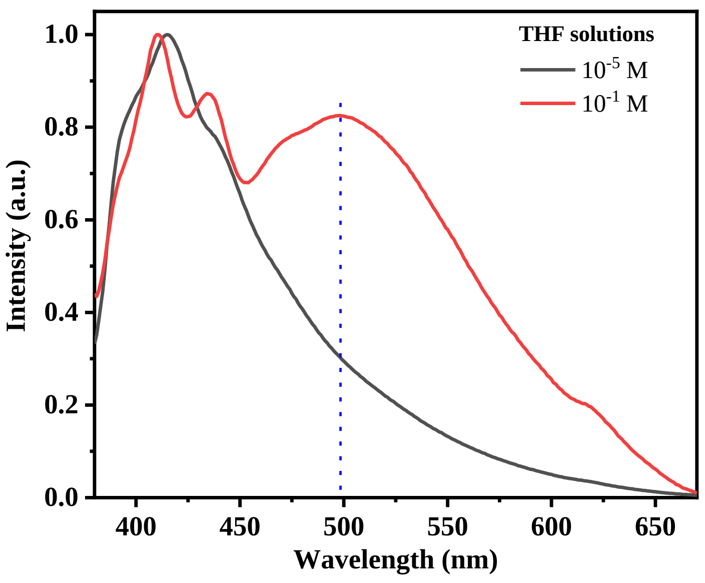

Figure S12. Normalized PL spectra of DDOP excited at 360 nm at variable concentrations in THF at room temperature.

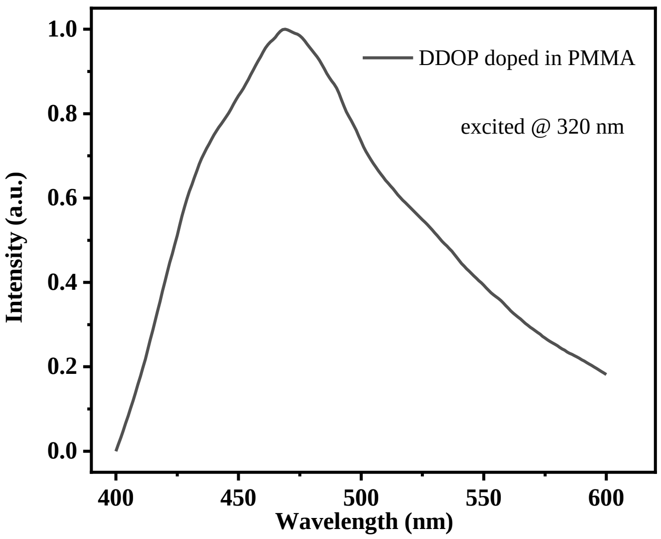

Figure S13. Normalized PL spectra of DDOP doped in PMMA matrix (5 wt.%) at room temperature in the atmosphere.

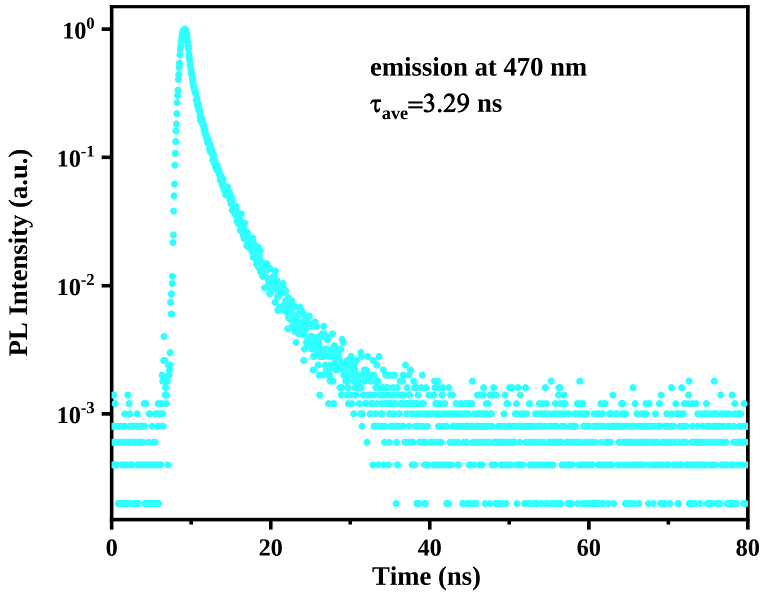

Figure S14. Lifetime decay profiles of DDOP pristine crystalline powders with emission peak at 470 nm at room temperature in the atmosphere.

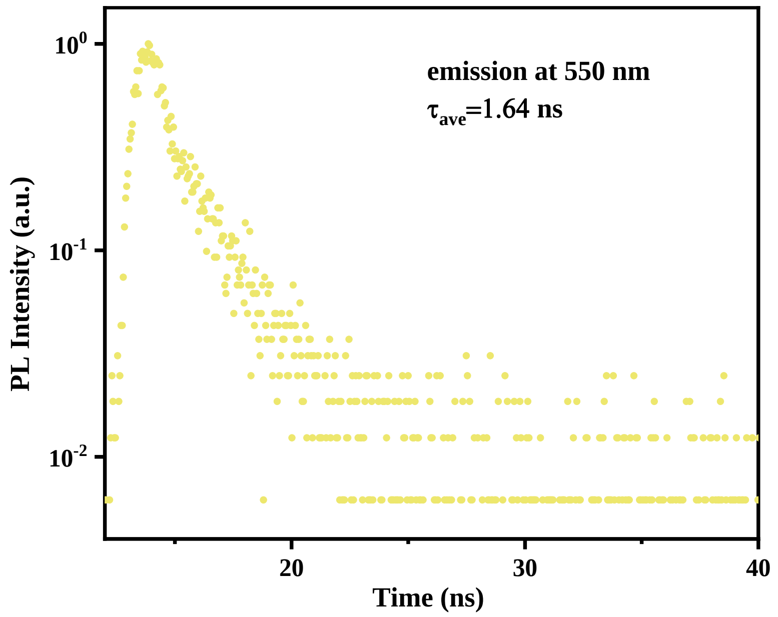

Figure S15. Lifetime decay profiles of DDOP pristine crystalline powders with emission peak at 550 nm at room temperature in the atmosphere.

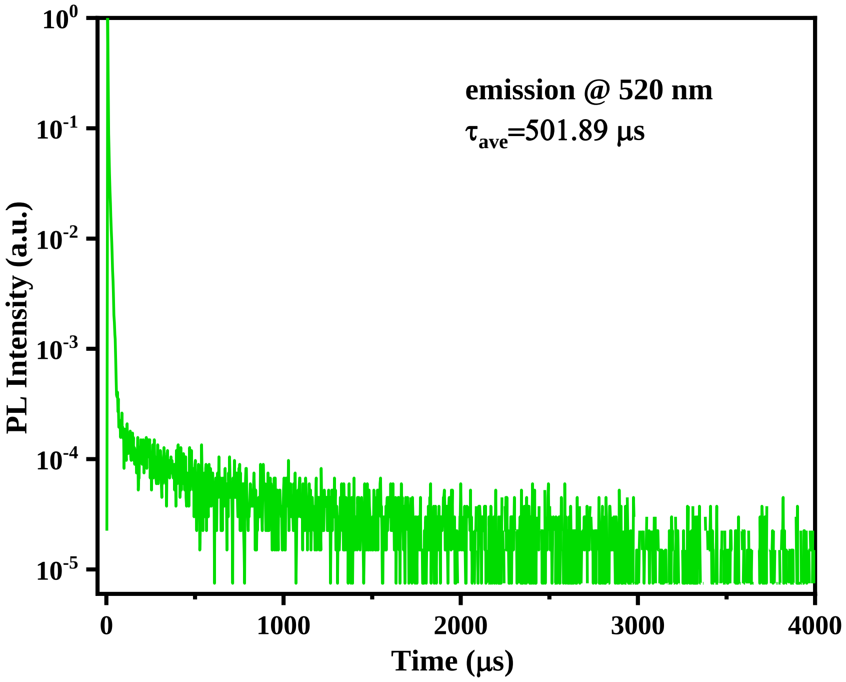

Figure S16. Lifetime decay profiles of DDOP pristine crystalline powders with emission peak at 520 nm at room temperature in the atmosphere.

Table S3. Lifetimes (τ) of DDOP pristine crystalline powders with emission peak at 470 nm and 550 nm at room temperature in the atmosphere.

	Excitation wavelength (nm)	Emission wavelength (nm)	Luminescence						
			τ_1	A_1 (%)	τ_2	A_2 (%)	τ_3	A_3 (%)	τ_{ave}
DDOP crystalline Powders	379	470	1.76 ns	54.51	5.12 ns	45.49	--	--	3.29 ns
	320	550	0.52 ns	26.03	2.03 ns	73.97	--	--	1.64 ns
	379	520	7.69 μs	50.35	166.12 μs	10.51	1227.8 μs	39.14	501.89 μs

The average lifetime was calculated according to $\tau_{ave}=\tau_1*A_1\ \%+\tau_2*A_2\ \%+\tau_3*\ A_3\ \%$, the calculated average lifetimes (τ_{ave}) were appeared in results and discussion.

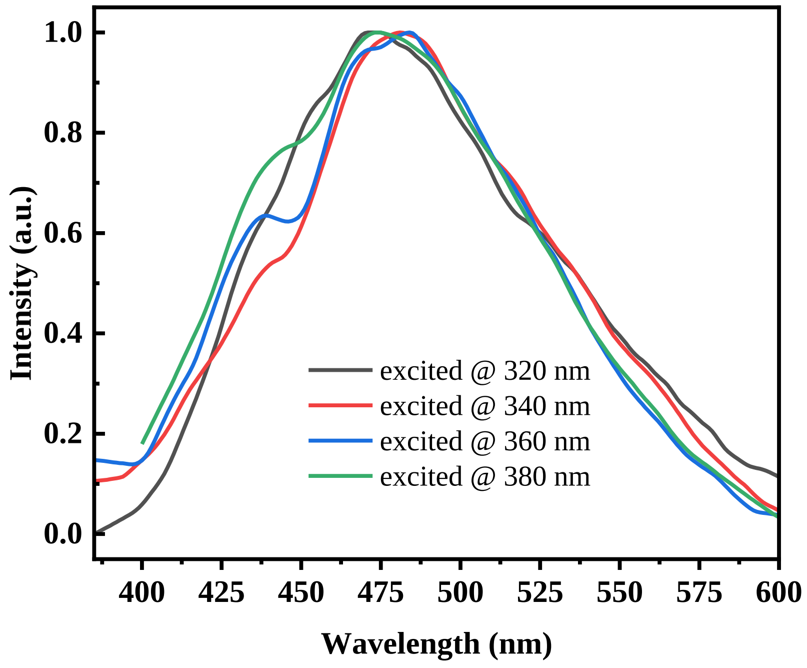

Figure S17. PL spectra of DDOP pristine crystalline powders excited by various excitation wavelengths at low temperature in liquid nitrogen.

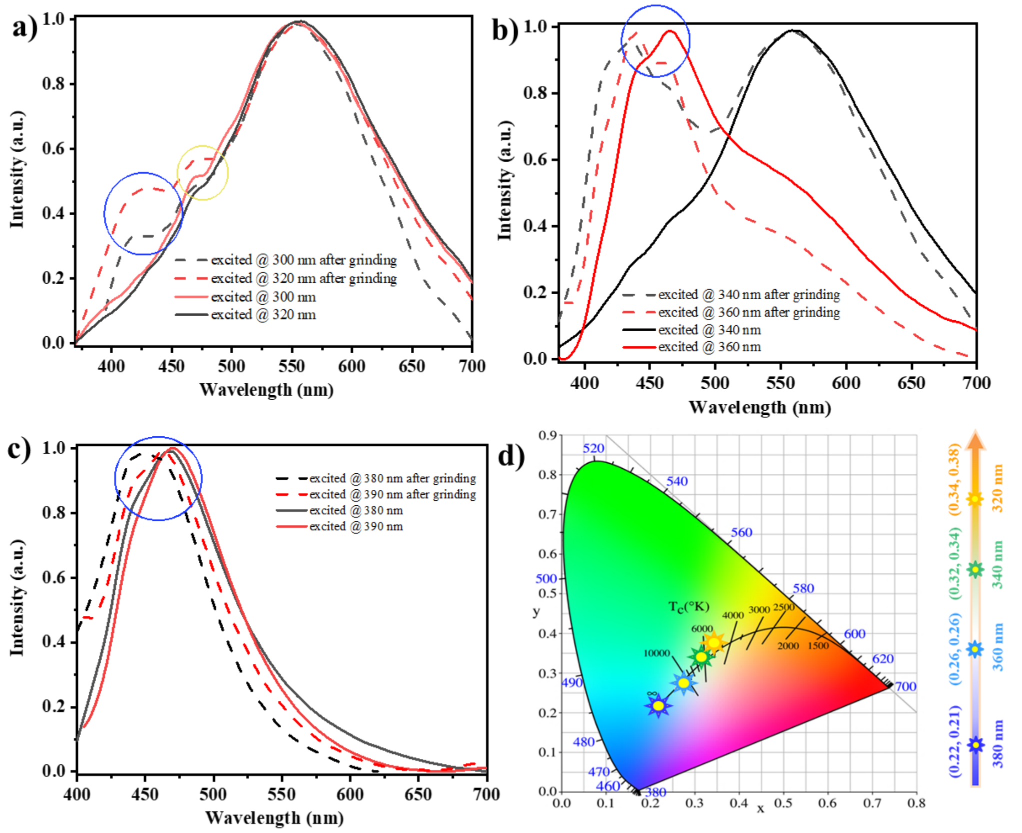

Figure S18. a)-c) Normalized PL spectra of DDOP pristine crystalline powders excited at different wavelengths before/after grinding at room temperature in the atmosphere; d) The calculated CIE coordinates of DDOP pristine crystalline powders under different excitation wavelengths.

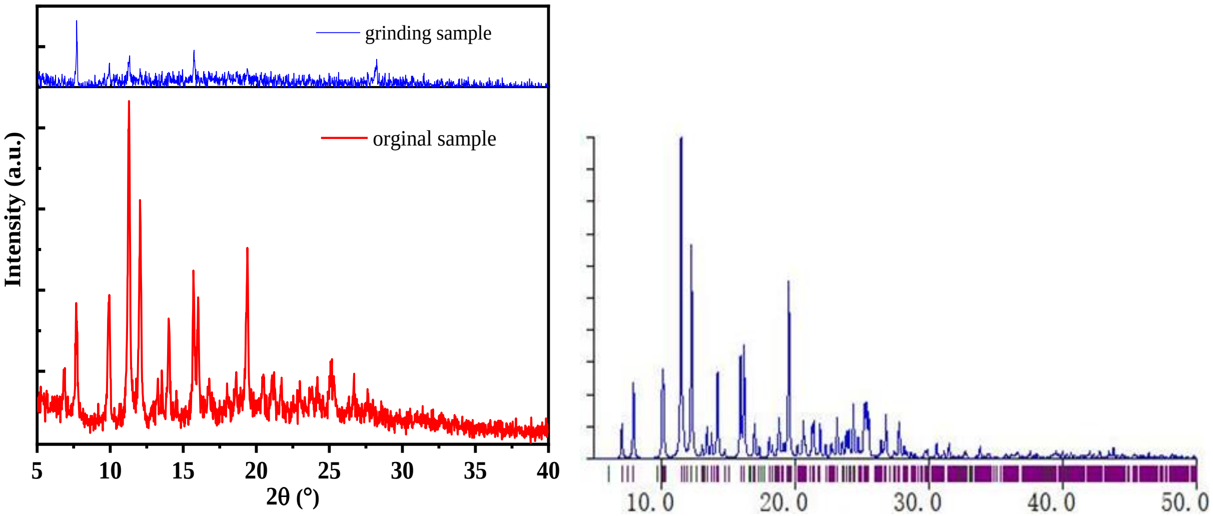

Figure S19. PXRD pattern of DDOP pristine crystalline powders before/after grinding (left) and the simulated diffraction pattern by Mercury 2020.1 (right).

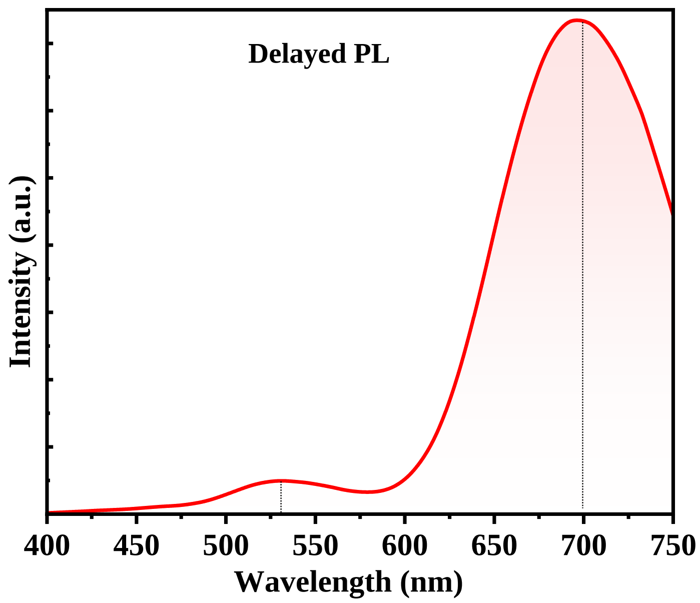

Figure S20. Delayed PL spectra for DDOP single crystal.

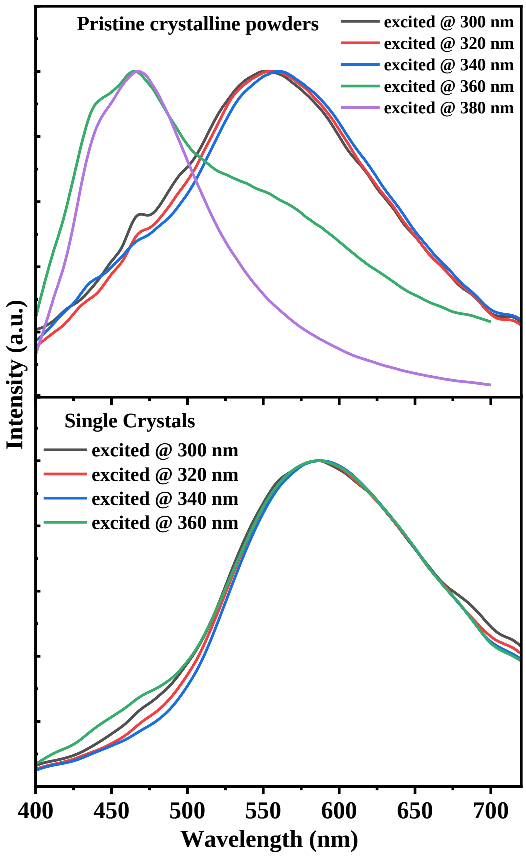

Figure S21. PL spectra of DDOP pristine crystalline powders and single crystals at different wavelengths.

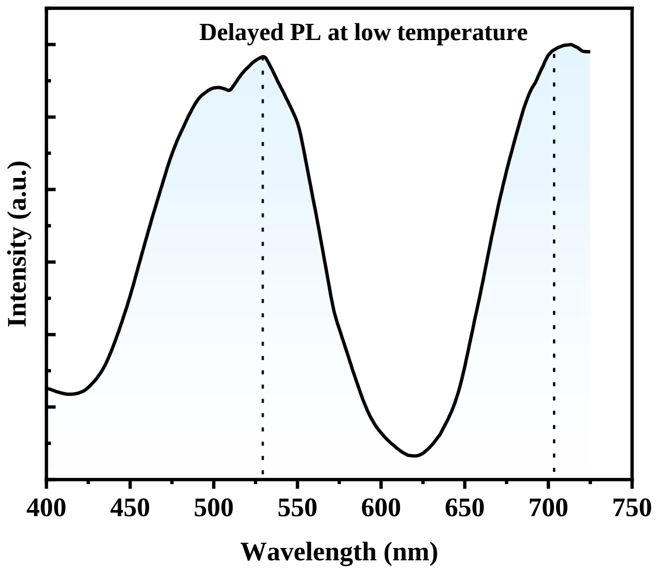

Figure S22. The delayed PL spectra at low temperature in liquid nitrogen of DDOP single crystal.

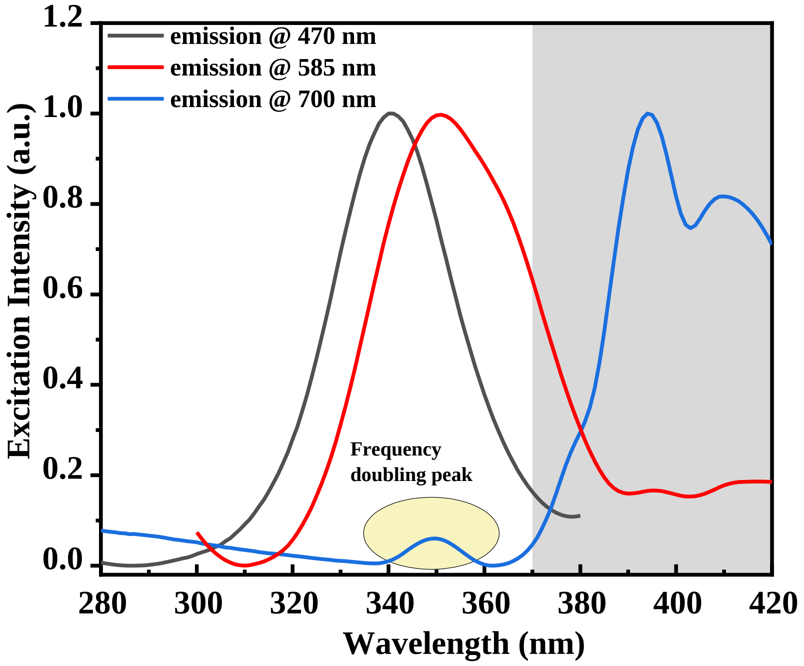

Figure S23. Excitation spectra of different emission wavelengths of DDOP single crystal.

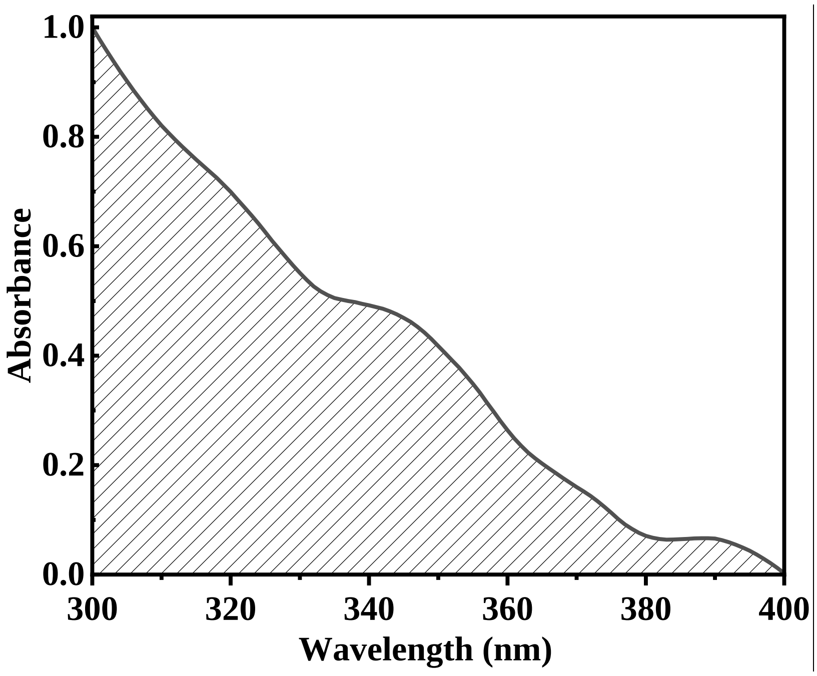

Figure S24. Normalized absorption spectra of DDOP single crystal.

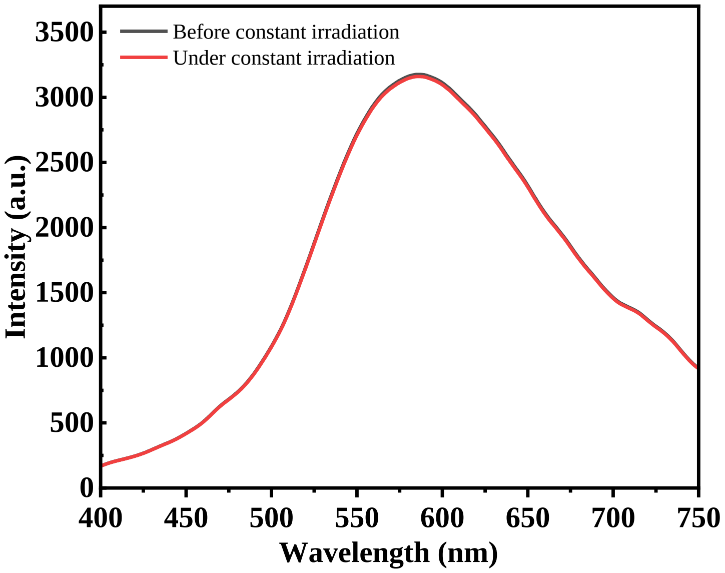

Figure S25. PL spectra of DDOP single crystals before/after the constant irradiation when excited at 320 nm.

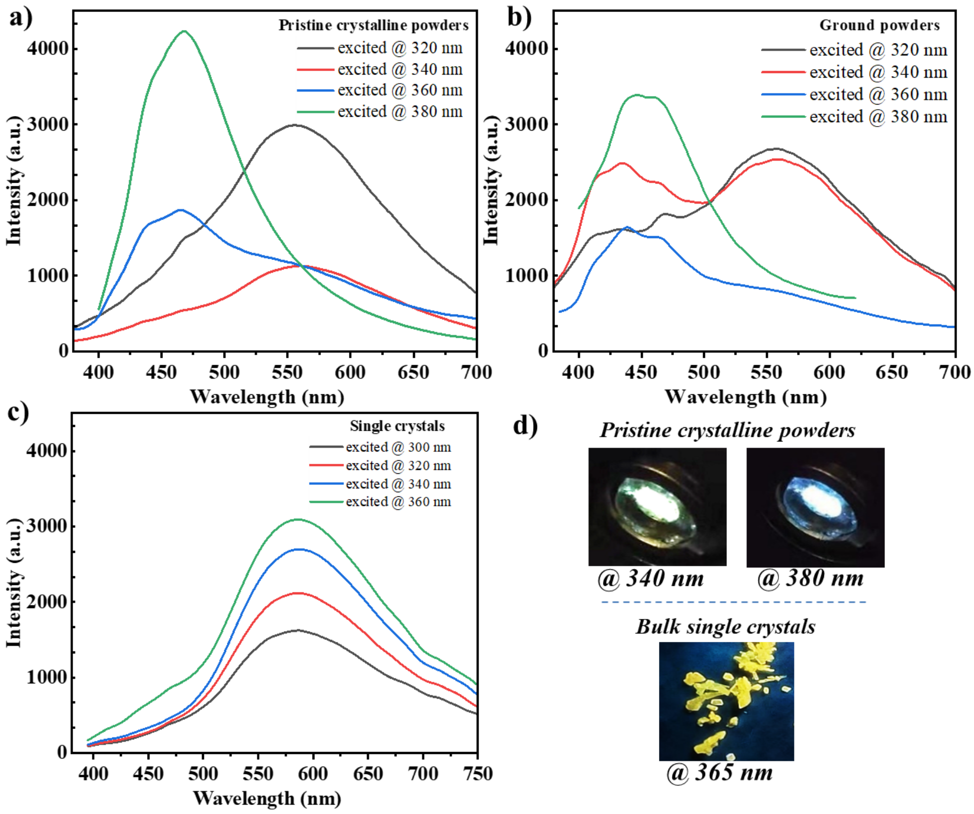

Figure S26. PL spectra excited at different wavelengths and the corresponding emissive images of DDOP. a) PL spectra of pristine crystalline powders; b) PL spectra of ground powders; c) PL spectra of single crystals; d) Emissive images of DDOP.

It can be observed that pristine crystalline powders exhibited yellow-dominated emission peaking at 550 nm with small shoulders at around 470 nm when excited at 320 and 340 nm. The intensity of emission peaks at 550 nm were close to 3000 and 1200 when excited at 320 and 340 nm, respectively (Figure S26a). In contrast, the main emission peaks were located at around 470 nm when excited at 360 and 380 nm. The intensity of emission peaks at 470 nm were more than 4200 and 1800 when excited at 320 and 340 nm, respectively (Figure S26a). As shown in Figure S26b, the emission peaks were slightly blue-shifted when excited at 360 and 380 nm. The intensities of main emission peaks were close to 3300 and 1600 when excited at 360 and 380 nm, respectively. The intensity of emission peaks at 550 nm were more 2600 when excited at 320 nm. Especially, under 340 nm excitation, almost white emission was observed with closely ratiometric blue and yellow emission intensities of around 2500. As shown in Figure S26c, DDOP single crystals presented consistent PL spectral shapes with varied emission intensity when excited at different wavelengths. The emission intensity was more than 3000 under 360 nm excitation.

Table S4. Lifetimes (τ) of DDOP single crystal with emission peak at 470, 585 nm, and 700 nm at room temperature in the atmosphere.

	Excitation wavelength (nm)	Emission wavelength (nm)	Luminescence						
			τ_1	A_1 (%)	τ_2	A_2 (%)	τ_3	A_3 (%)	τ_{ave}
DDOP single crystal	320	470	0.51 ns	24.28	2.18 ns	75.72	--	--	1.77 ns
		530	8.97 µs	50.35	87.18 µs	10.51	1177.43 µs	39.14	474.53 µs
		585	4.05 µs	15.00	22.01 µs	60.57	130.06 µs	24.43	45.71µs
		700	26.40 µs	16.36	411.82 µs	18.40	2952.44 µs	65.24	2006.27 µs

Table S5. The PLQYs of DDOP pristine crystalline powders excited at 380 nm and 320 nm, as well as single crystals excited at 360 nm at room temperature in the atmosphere.

	Aggregation state	Excitation wavelength	PLQY
DDOP	Pristine crystalline powder	380 nm	8.3%
	Pristine crystalline powder	320 nm	5.8%
	Single crystal	380 nm	9.8%

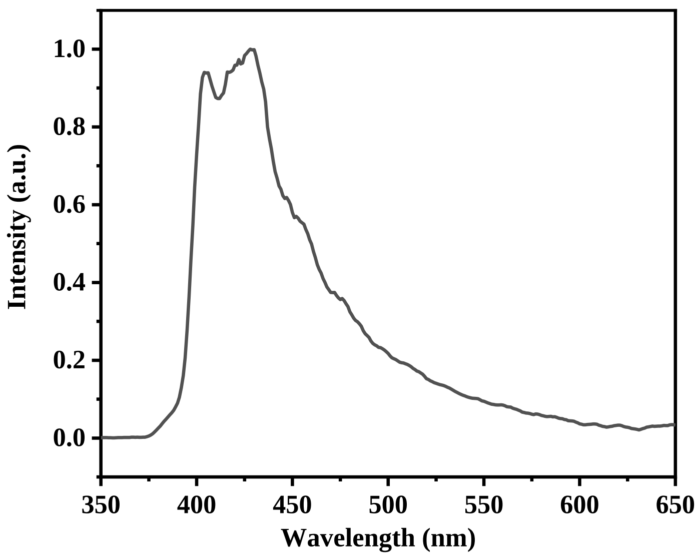

Figure S27. Normalized phosphorescence spectra of DDOP in 2-methyltetrahydrofuran (5.0×10^{-5} M) at low temperature in liquid nitrogen excited at 320 nm.

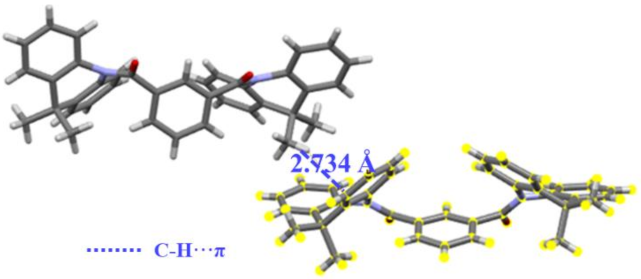

Figure S28. C-H···π intermolecular interactions in Dimer 3 of DDOP single crystals.

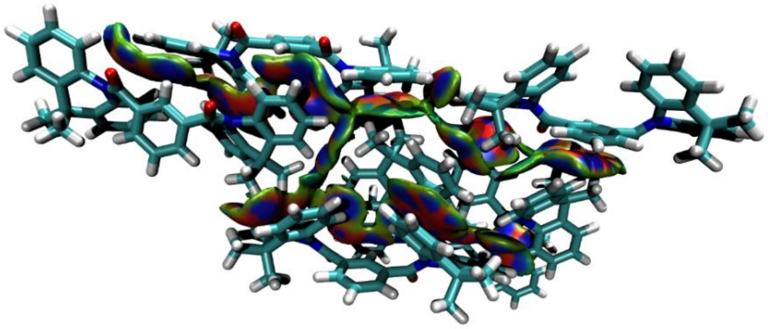

Figure S29. The calculated molecular interactions in single crystal cell of DDOP.

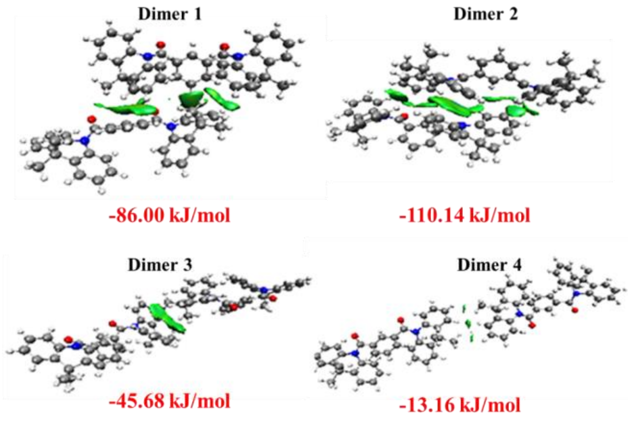

Figure S30. The calculated molecular interactions (green isosurface) in different dimers of DDOP single crystal.

Table S6. Single crystal data for DDOP cultivated by the solvents of DCM/MeOH.

Name		DDOP
Formula		$C_{38}H_{32}N_2O_2$
Crystal system		Monoclinic
Space group		C2/c (No. 15)
	a	28.839(3)
Cell Lengths (Å)	b	9.3212(11)
	c	25.728(3)
	α	90
Cell Angles (°)	β	120.370(3)
	γ	90
Cell volume (Å^3)		5967.0(12)
Z		8
Density (g/cm^3)		1.437
F(000)		2640
h_{max}, k_{max}, l_{max}		38, 12, 28
CCDC Number		2045734

Table S7. Single crystal data before/after constant irradiation for DDOP.

**Name**		**DDOP (Before constant irradiation)**	**DDOP (After constant irradiation)**
**Formula**		$C_{38}H_{32}N_2O_2$	
**Crystal system**		Monoclinic	
**Space group**		C2/c (no. 15)	
**Cell Lengths (Å)**	**a**	28.839(3)	28.7376(18)
	**b**	9.3212(11)	9.2916(5)
	**c**	25.728(3)	25.6879(15)
**Cell Angles (°)**	**α**	90	90
	**β**	120.370(3)	120.4660(10)
	**γ**	90	90
**Cell volume (Å^3)**		5967.0(12)	5912.1(6)
**Z**		8	8
**Density (g/cm^3)**		1.437	1.426
**F(000)**		2640	2596
h_{max}, k_{max}, l_{max}		38, 12, 28	38, 12, 33

No obvious changes in the molecular structure and conformation have been observed for the single crystal before and after UV illumination, confirming their photo-stability. The unit cell parameters only vary within 0.1 Å, which is considered a normal experimental error.

Table S8. Decomposition for interaction energy in different dimers of DDOP single crystal.

	Electrostatic (kJ/mol)	Repulsion (kJ/mol)	Dispersion (kJ/mol)	Total (kJ/mol)
Dimer 1	-9.73	48.90	-125.17	-86.00
Dimer 2	-2.62	54.67	-162.19	-110.14
Dimer 3	-2.57	17.99	-61.08	-45.68
Dimer 4	0.13	3.46	-16.75	-13.16

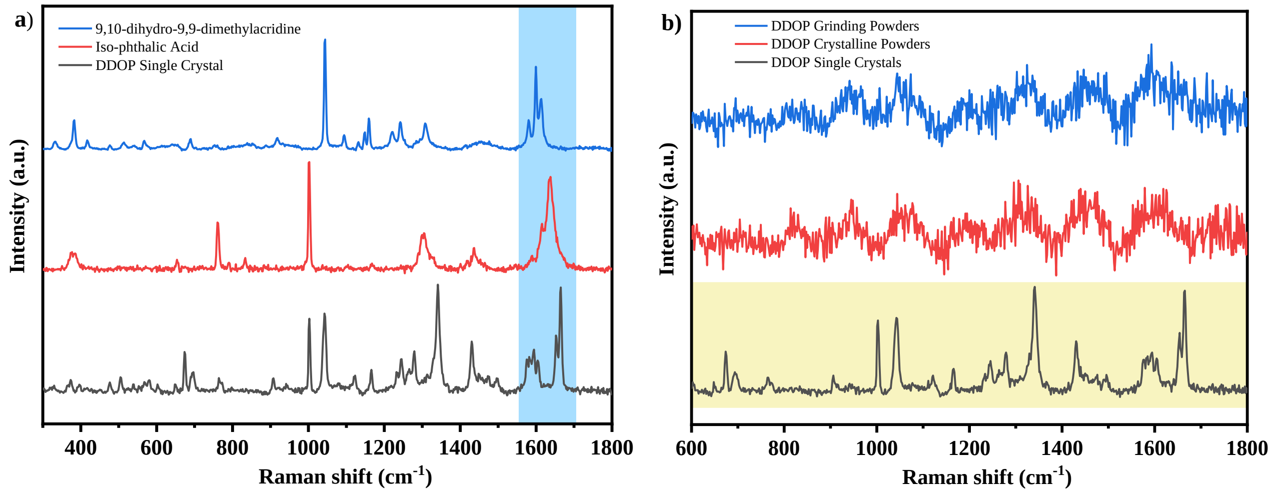

Figure S31. a) Fragment of Raman spectra of DDOP single crystals, DMAC (9,10-dihydro-9,9-dimethylacridine), and isophthalic acid; b) Fragment of Raman spectra of DDOP single crystal and powders before/after grinding.

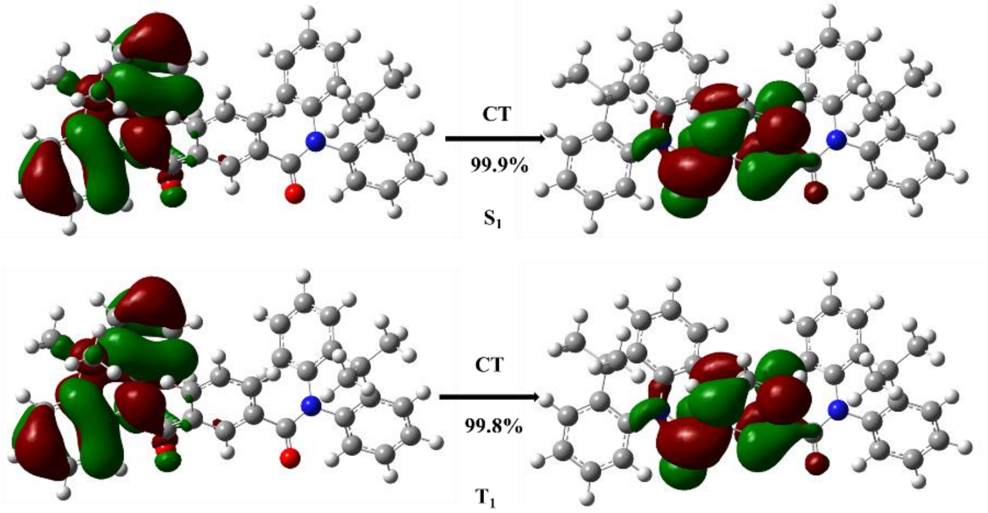

Figure S32. The NTOs for the monomer of DDOP.

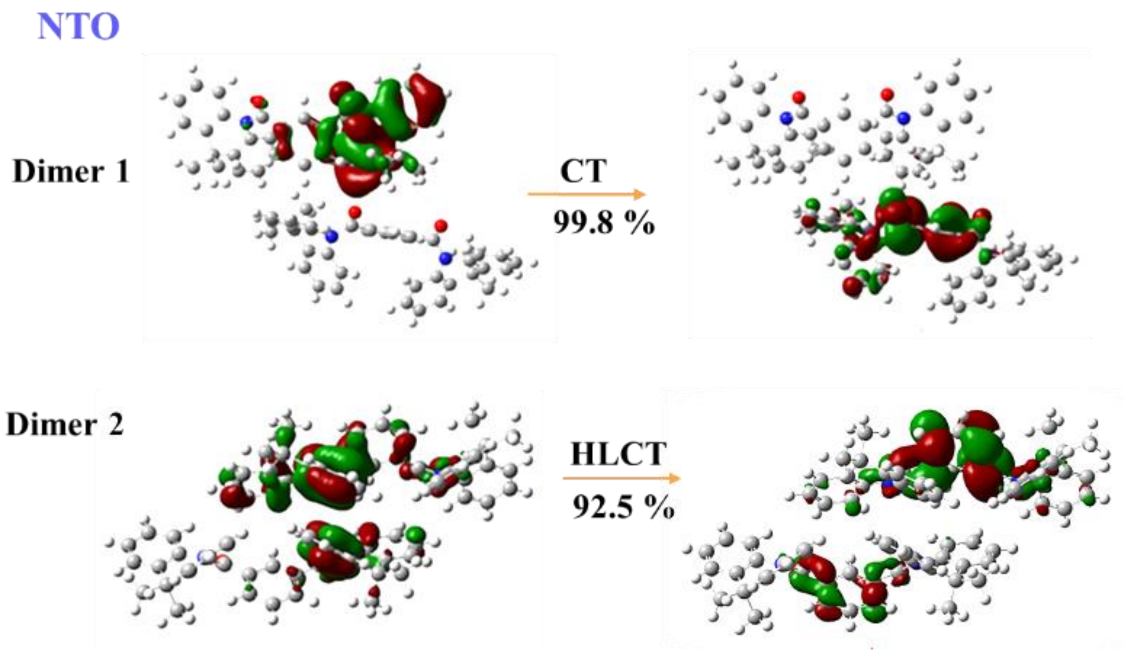

Figure S33. The NTOs for Dimer 1 and Dimer 2 of DDOP.

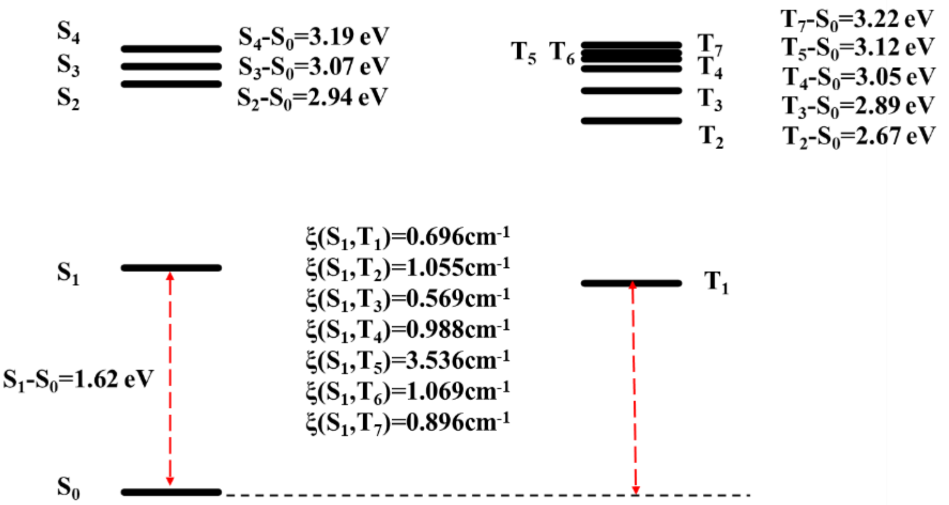

Figure S34. Calculated energy diagrams for the monomer based on DDOP single crystal.

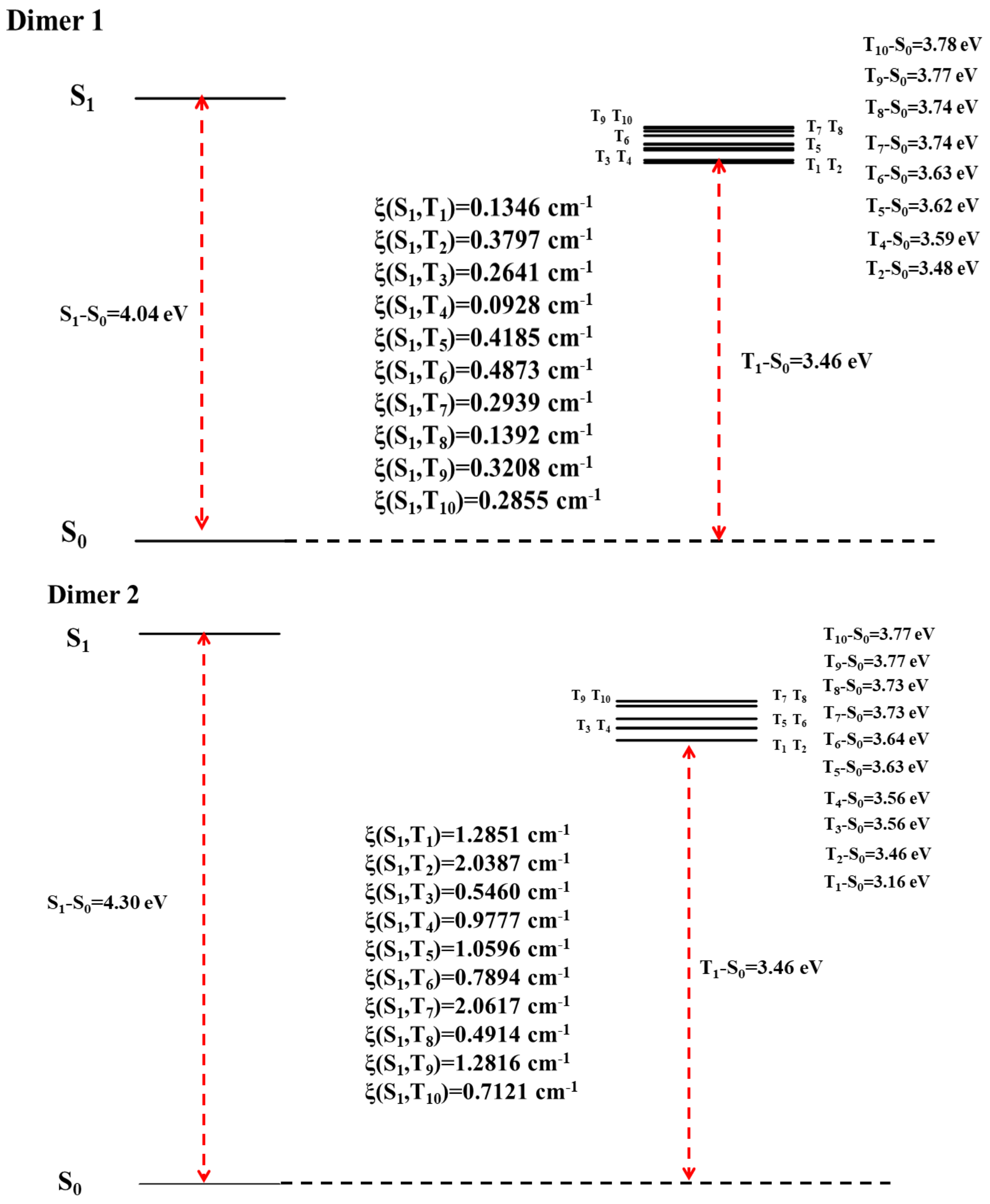

Figure S35. Calculated energy diagrams for Dimer 1 and Dimer 2 based on DDOP single crystal.
